# Exosomes in HPV-Associated Cancers: From Biomarkers to Engineered Therapeutics

**DOI:** 10.3390/cancers17203386

**Published:** 2025-10-21

**Authors:** Muharrem Okan Cakir, Melis Selek, Betul Yilmaz, Mustafa Ozdogan, G. Hossein Ashrafi

**Affiliations:** 1 School of Life Sciences, Pharmacy and Chemistry, Kingston University London, London KT1 2EE, UK; m.okan@kingston.ac.uk; 2 School of Medicine, Koc University, Sarıyer, Istanbul 34450, Turkey; mselek22@ku.edu.tr; 3 Department of Biochemistry, School of Medicine & Genetic and Metabolic Disease Research and Investigation Center, Marmara University, Istanbul 34854, Turkey; betulkarademir@marmara.edu.tr; 4 Department of Biochemistry, School of Medicine, Recep Tayyip Erdogan University, Rize 53100, Turkey; 5 Division of Medical Oncology, Memorial Hospital, Antalya 07050, Turkey; mustafa.ozdogan@medstar.com.tr

**Keywords:** exosomes, extracellular vesicles, human papillomavirus (HPV), liquid biopsy, biomarkers, cervical cancer, head and neck squamous cell carcinoma (HNSCC)

## Abstract

**Simple Summary:**

Human papillomavirus (HPV) is the leading cause of cervical cancer and a significant contributor to several other cancers, including head and neck malignancies. Recent research highlights the role of exosomes, small extracellular vesicles involved in cell communication, as potential diagnostic and therapeutic tools in HPV-associated cancers. Exosomes can carry molecular information reflective of tumor behavior and HPV status, making them ideal candidates for non-invasive liquid biopsy. Additionally, they are being investigated for their roles in vaccine delivery, immune stimulation, and overcoming treatment resistance. This review explores how exosomal content (such as miRNAs, proteins, and lncRNAs) may be used to monitor disease progression, predict therapy response, and even serve as treatment vehicles. We also discuss challenges such as lack of standardization and the need for large cohort validation, and we highlight opportunities for clinical translation in the coming years.

**Abstract:**

Background/Objectives: Human papillomavirus (HPV) is the main causative agent of cervical cancer and contributes to a significant proportion of other anogenital and oropharyngeal malignancies. The need for better biomarkers and therapeutic approaches in HPV-associated cancers has drawn attention to exosomes, small extracellular vesicles known for their stability, biomolecule transport capabilities, and role in cell-to-cell communication. Methods: This review comprehensively evaluates recent literature on the diagnostic, prognostic, and therapeutic applications of small extracellular vesicles, particularly exosomes, in HPV-related cancers. It analyzes findings on exosomal nucleic acids, proteins, and long non-coding RNAs, as well as engineered exosome-based therapies. Results: Exosomal miRNAs (e.g., miR-204-5p, miR-99a-5p, miR-21), proteins (e.g., glycolytic enzymes, HSP90), and lncRNAs (e.g., HOTAIR, DLEU1) have emerged as promising biomarkers for disease detection and monitoring. Exosomal cargo actively participates in HPV-related tumor progression. For example, miRNAs such as miR-21 and miR-146a modulate immune cell polarization and inflammatory signaling, while lncRNAs like HOTAIR promote oncogenic transcriptional programs. Exosomal proteins including HSP90 and ANXA1 facilitate extracellular matrix remodeling and immune evasion, thereby influencing tumor growth and metastasis. In HPV-positive head and neck and cervical cancers, exosomal cargo reflects HPV status, tumor progression, and treatment response. Therapeutic studies demonstrate the utility of exosomes in vaccine delivery, immune modulation, and drug delivery systems, including the use of PROTACs. However, clinical translation faces barriers including isolation protocol standardization, biomarker validation, and scalable production. Conclusions: Exosomes hold great promise for integration into diagnostic and therapeutic workflows for HPV-related cancers. Future research should focus on resolving standardization issues, validating biomarkers in diverse cohorts, and optimizing engineered exosome platforms for targeted therapy.

## 1. Introduction

Being the primary causative agent for nearly all cervical malignancies and a high fraction of other anogenital and oropharyngeal cancers such as head and neck, vaginal, vulvar, and penile cancers, Human Papillomavirus (HPV) represents a serious global health burden [1]. Globally, cervical cancer presents as the 2nd most common cancer in women and is estimated to have caused 350,000 deaths with 660,000 new cases in 2022. It also places 4th in terms of mortality. Low-to-middle-income countries are the most affected, as they lack sufficient screening, prevention, and treatment options [2,3]. From data as of August 2022, a total of 21,800 cancer cases for women and 16,000 cancer cases for men each year are thought to be due to HPV [1]. It takes a long time, often decades, for a person infected with HPV to develop cancer, and there is no sure way of knowing who will undergo malignant transformation [1].

Clinically, HPV-positive cancers, particularly in the cervix and oropharynx, tend to present at a younger age and often have a better prognosis than their HPV-negative counterparts, though they may differ in patterns of metastasis and treatment response [4,5]. At the molecular level, HPV oncogenes E6 and E7 inactivate tumor suppressors p53 and pRb, driving uncontrolled cell cycle progression, genomic instability, and immune evasion [6]. Prophylactic vaccination with bivalent, quadrivalent, or nonavalent formulations has demonstrated high efficacy in preventing infection by high-risk HPV types, leading to a measurable decline in precancerous lesions where coverage is high [7,8,9].

In recent years, extracellular vesicles (EVs), particularly exosomes, have gathered attention in the field of oncology as they were demonstrated to play a key role in intercellular signaling and to mirror the functional and disease-related status of their parent cells [10]. Exosomes are vesicles with diameters ranging from 30 to 150 nm, and they can be secreted by many cell types, including cancer cells [11]. Exosomes are a subtype of small extracellular vesicles (sEVs), originating from endosomal multivesicular bodies and distinct from other EVs such as microvesicles and apoptotic bodies. In this review, we use the term ‘exosomes’ to specifically refer to sEVs of endosomal origin, unless otherwise indicated [12]. Exosomes can be detected in various bodily fluids like saliva, blood and urine [13,14]. These vesicles can carry a diverse cargo of biomolecules with different functions, including nucleic acids such as DNA, RNA, miRNAs, lncRNAs, proteins, and lipids. The cargo can also be transferred to recipient cells, which influences their function [15]. This mechanism of cargo donation can potentially contribute to cancer progression, metastasis, and even drug resistance [16].

While established diagnostic tools such as Pap smears, HPV DNA testing, and detection of circulating tumor nucleic acids (cfDNA, ctRNA) have improved early detection, they are limited by sensitivity in early-stage lesions, inability to provide real-time monitoring, and lack of comprehensive molecular profiling. Exosomes, by contrast, are stable carriers of diverse biomolecules and can reflect both viral and host molecular states [17,18,19]. Exosomes have valuable and unique properties that make them promising non-invasive biomarkers such as being stable in the circulation, protecting the cargo from degradation with a lipid bilayer, and being able to pass cellular barriers [11]. Their cargo offers a dynamic and integrative view of tumor biology, making them promising tools for earlier detection, longitudinal monitoring, and therapeutic guidance in HPV-related malignancies. Liquid biopsy can be used as a less invasive alternative to the traditional tissue biopsy and allows to monitor disease progression and response to treatment in real time. It can also be useful for early detection of malignancies [13,20]. It allows clinicians to generate a characteristic molecular picture of cancer. Exosomes-based liquid biopsy is a candidate with high potential in the field of HPV-related cancers, as exosomal content is shown to change in various ways with respect to the HPV infection status in different types of cancers [21]. The dynamic changes in the tumor microenvironment (TME) can be monitored through the exosomal cargo that closely mirror the ongoing changes [22].

Compared with exosomes in other cancers such as prostate or breast cancer, HPV-associated cancer exosomes often display cargo patterns shaped by viral oncoproteins E6 and E7, which reprogram host transcription, splicing, and immune signaling. This viral regulation can lead to enrichment of immune-modulatory miRNAs, altered metabolic enzymes, and viral RNA species not present in non-viral cancers, underscoring their potential specificity as biomarkers [21,23,24,25]

In addition to their potential use in diagnosis and prognostic determination, exosomes are also investigated as novel tools in cancer therapeutics. Their natural compatibility with the body, low potential to trigger immune responses, and ability to transport functional biological cargo make exosomes good vehicles for precision therapies, vaccine platforms, and standalone treatment approaches [26]. This review aims to present a comprehensive overview of the current understanding and future prospects of exosomes in HPV-associated malignancies by exploring their applications as biomarkers in liquid biopsy for detection, prognosis, and monitoring, as well as their promising therapeutic potential. This review is, to our knowledge, the first to integrate a synthesis of HPV-specific exosomal biomarkers with an in-depth discussion of engineered exosome-based therapies, including emerging strategies such as PROTAC delivery and ligand-modified targeting, alongside recent clinical and preclinical studies.

## 2. Exosomal Molecular Mechanisms in HPV+ Cancers

### 2.1. Immune Modulation and Evasion

**Figure 1:**
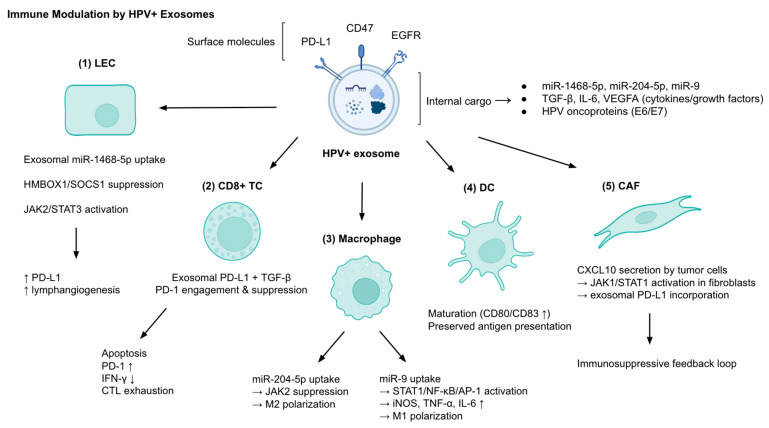
Immune modulation by HPV^+^ exosomes. HPV^+^ exosomes carry surface molecules (PD-L1, CD47, EGFR) and internal cargo (miR-1468-5p, miR-204-5p, miR-9, TGF-β, IL-6, VEGFA, HPV oncoproteins E6/E7), which interact with multiple cell types in the tumor microenvironment (TME). In lymphatic endothelial cells (LECs), miR-1468-5p uptake suppresses HMBOX1/SOCS1 and activates JAK2/STAT3, driving PD-L1 expression and lymphangiogenesis. In CD8^+^ T cells (TCs), exosomal PD-L1 and TGF-β induce PD-1 engagement, apoptosis, IFN-γ reduction, and cytotoxic T lymphocyte (CTL) exhaustion. In macrophages, miR-204-5p promotes M2 polarization via JAK2 suppression, while miR-9 favors M1 polarization via STAT1/NF-κB/AP-1 activation and increased iNOS, TNF-α, and IL-6. Dendritic cells (DCs) exposed to HPV^+^ exosomes undergo maturation (CD80/CD83↑) with preserved antigen presentation, unlike HPV– exosomes. Cancer-associated fibroblasts (CAFs) are reprogrammed through CXCL10–JAK1/STAT1 signaling to incorporate PD-L1 into their own exosomes, creating an immunosuppressive feedback loop. Abbreviations: CAF, cancer-associated fibroblast; CTL, cytotoxic T lymphocyte; DC, dendritic cell; EGFR, epidermal growth factor receptor; HPV, human papillomavirus; IFN-γ, interferon-γ; IL, interleukin; iNOS, inducible nitric oxide synthase; JAK, Janus kinase; LEC, lymphatic endothelial cell; NF-κB, nuclear factor kappa B; PD-1, programmed cell death protein 1; PD-L1, programmed death-ligand 1; SOCS1, suppressor of cytokine signaling 1; STAT, signal transducer and activator of transcription; TC, T cell; TGF-β, transforming growth factor-β; TEX, tumor-derived exosome; VEGFA, vascular endothelial growth factor A.

The tumor immune microenvironment is regulated by the HPV-associated exosomes through the actions of their diverse molecular cargo and cellular interactions, with the aim of achieving immune evasion and immune response orchestration (Figure 1). In HPV-positive cervical and head and neck cancers, proteomic and transcriptomic profiles of exosomes exhibited enrichment in immune-related pathways, namely immune system signaling, neutrophil degranulation, and immune suppression. A strong immunomodulatory signature (EVsig) from HPV(+) EVs correlated with increased infiltration of CD4+ T cells, macrophages, neutrophils, and dendritic cells. This effect did not extend to cytotoxic T cells and B cells. Genes such as TGFB1, PD-L1 (CD274), and NT5E/CD73, which have immunosuppressive roles, showed augmented expression, further supporting an immune invasive TME [27,28]. In addition, exosomes derived from cervical cancer enriched in miR-1468-5p were taken up by lymphatic endothelial cells (LECs), where they suppressed HMBOX1 and the downstream protein SOCS1, activating the JAK2/STAT3 pathway. The result was increased PD-L1 expression and lymphangiogenesis. Impaired CD8+ T cell activity that is characterized by elevated PD-1 expression, apoptosis, and reduced IFN-γ production, was observed. This state provides tumor escape from immunity, and high serum miR-1468-5p levels correlated with poor survival outcomes [29].

Several exosomal components have been mechanistically implicated in facilitating immune evasion. Likewise, HPV(+) exosomes carry viral and immune-related transcripts such as PD-L1, IL6, and VEGFA [30,31]. And CD47, a “don’t eat me” signal, is uniquely expressed on the surface of exosomes, leading to reduced phagocytic clearance by monocytes [31,32]. CD47 packaging into exosomes is regulated by the EGFR/STAT3 pathway via the RAB31 transporter, illustrating that a dual mechanism of immune evasion, transcriptional and vesicular, is present [32]. In addition to the properties adopted by tumor cells, exosome-mediated education of fibroblasts has been observed: HPV(+) cervical cancer cells secrete CXCL10, which activates PD-L1 expression in fibroblasts via JAK1/STAT1 signaling. The induced PD-L1 is incorporated into fibroblast-derived exosomes and not secreted freely, creating a loop of immunosuppressive feedback [33].

HPV(+) and HPV(−) tumors are further differentiated through the exosomal effects on dendritic cells (DCs). HPV(+) and HPV(−) exosomes both suppress T cell proliferation and induce the apoptosis of CD8+ T cells, but only HPV+ exosomes promote DC maturation which is marked by the upregulation of CD80 and CD83. HPV- exosomes, on the other hand, inhibit the co-stimulatory and antigen-processing properties of DCs, resulting in impaired T cell priming. HPV+ HNSCC are thought to respond better to immunotherapy with better prognosis overall compared to HPV- tumors, which could be attributed to the partially remaining antigen presentation mechanism [25]. Another factor relating to better therapy response in HPV(+) tumors could be that HPV(−) exosomes are enriched in tumor-promoting and immunosuppressive proteins such as MUC-1, HLA-DRA, MUC-4, and MUC-16, which give protection to tumor cells against NK cell-mediated lysis. This is not a function observed in HPV(+) exosomes [31]. Moreover, in head and neck cancer (HNC), exosomes from HPV(+) and HPV(−) cells were found to carry distinct mRNA and miRNA profiles reflecting their origin: HPV(+) exosomes were enriched in HPV16 E6/E7, EGFR, TP53, and miR-205-5p, while HPV(−) exosomes showed higher levels of immunosuppressive mRNAs like FAS and DPP4, and miR-1972, which can interfere with antigen processing pathways [34].

Cancer-associated fibroblast (CAF) behavior is also manipulated by exosomes. Lower CAF infiltration, associated with better clinical outcome, is observed in HPV+ head and neck squamous cell carcinomas (HNSCC). In HPV+ tumors, TGF-β-induced fibroblast activation is inhibited through the action of the secreted miR-9-5p enriched exosomes that target NOX4, dampening formation of ROS and progression of Smad2/3 signaling and resulting in suppressed myofibroblast transformation. This maintains a less fibrotic, more immune-permissive microenvironment [35].

The exosome production of immune cells can also be modulated in the TME by the HPV infection. In HPV-related penile squamous cell carcinoma (PSCC), regulatory T cells secrete exosomes containing lncRNAs that interact with miR-619-3p, which leads to increased expression of MAL (Myelin and Lymphocyte protein) in cytotoxic T cells that results in their apoptosis. This mechanism of Treg derived exosomes results in impaired anti-tumor immunity, promoting tumor growth via reduced CTL infiltration [36].

Exosomes also affect macrophage activity and polarization in the TME. Exosomes derived from HPV16 E7-pulsed dendritic cells (DCs) demonstrated inhibition of macrophage migration, inflammation and M1 polarization. This resulted in promoting an M2-like anti-inflammatory phenotype both in vitro and in vivo. In DCs silenced for catalase 2 (CAT2), the observed effect was opposite: macrophage migration was enhanced with polarization towards M1, which suppressed tumor growth in an HPV cervical cancer mouse model. Notably, the different effects of E7-pulsed and CAT2-silenced DCs were mediated by their exosomes, suggesting a reciprocal regulation mechanism modifying tumor-associated macrophage behavior in HPV-associated CC [37]. Additionally, miR-204-5p, located in exosomes released from HPV16 E6-positive cervical cancer cells, was found to directly suppress JAK2 in the recipient macrophages, resulting in polarization toward the anti-inflammatory M2 phenotype [38]. Some studies present contrasting evidence indicating that HPV(+) exosomes may promote M1 polarization, challenging the notion that they only drive macrophages toward an M2 phenotype. For example, exosomes from HPV+ HNSCC and patient-derived HPV(+) tumors increased the expression of M1 markers (iNOS, TNF-α, IL-6) while reducing M2 markers (CD163, IL-10). These actions were largely mediated by the transfer of miR-9 and consequent activation of the transcription factors STAT1, NF-κB, and AP-1. The M1-skewing effects appear to be driven primarily by exosomal RNA cargo rather than surface proteins, highlighting the transcriptomic influence vesicles have in immune reprogramming [39,40]. Additional studies show exosomal involvement in TME shaping is greatly carried out through the selective packaging of immune-modulatory RNAs. EVs from HPV-transformed keratinocytes (K16 and K38 cells) displayed different mRNA signatures for inflammatory chemokines like CXCL10 (upregulated in K16 EVs), CCL2 (upregulated in K38 EVs), while lacking others (IL-1α/β downregulated in K16 EVs, CCL27/CTACK and CXCL3/Gro-γ lacking in both), indicating cargo selection as a strategy to modulate the immune response [41].

Taken together, these findings illustrate that HPV(+) exosomes can act in various ways to modulate the immune response through effects on different cell types (Table 1). They can suppress cytotoxic immunity, promote immune tolerance, and preserve or inhibit elements of signaling, leading to altered activation and polarization patterns. These may explain the paradox of better prognosis yet persistent immune evasion in HPV-driven cancers. The dual behavior of enhancing certain immune functions while disabling others highlights the complex but strategic role exosomes play in immunity of HPV-related tumor development.

### 2.2. EMT and Metastasis

**Figure 2:**
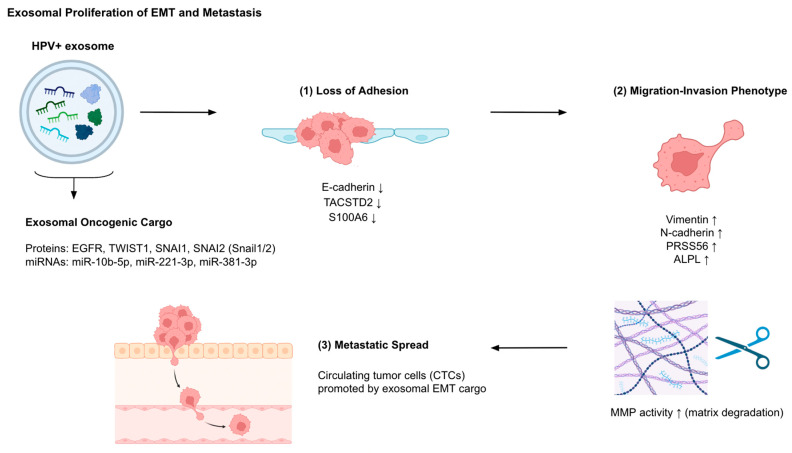
Exosomal promotion of epithelial–mesenchymal transition (EMT) and metastasis in HPV^+^ cancers. HPV^+^ exosomes deliver oncogenic cargo including proteins (EGFR, TWIST1, SNAI1, SNAI2) and miRNAs (miR-10b-5p, miR-221-3p, miR-381-3p), which collectively drive EMT progression. (1) Loss of adhesion is marked by reduced expression of epithelial junctional proteins (E-cadherin, TACSTD2, S100A6). (2) Recipient cells acquire a migration–invasion phenotype with increased mesenchymal markers (Vimentin, N-cadherin) and upregulated pro-metastatic proteins (PRSS56, ALPL), alongside matrix metalloproteinase (MMP) activity that degrades extracellular matrix. (3) These alterations promote metastatic spread via the generation of circulating tumor cells (CTCs). Abbreviations: ALPL, alkaline phosphatase; CTC, circulating tumor cell; EGFR, epidermal growth factor receptor; EMT, epithelial–mesenchymal transition; HPV, human papillomavirus; MMP, matrix metalloproteinase; PRSS56, serine protease 56; SNAI1/2, Snail1/2; TACSTD2, tumor-associated calcium signal transducer 2.

Exosomes from HPV-associated cancer cells facilitate epithelial–mesenchymal transition (EMT) and metastasis with protein and RNA cargo (Figure 2) (Table 1). Proteomic profiling of HeLa-derived extracellular vesicles (EVs) showed significant upregulation of proteins such as PRSS56, ALPL, and NPTX1, which were enriched in cell adhesion pathways, whereas downregulated proteins including ITGB4, TACSTD2, and S100A6 were associated with cell–cell adhesion, apoptotic regulation, and migration. These changes suggest a shift toward a pro-metastatic phenotype by disrupting intercellular attachment and enhancing motility [27]. In HN12-derived EVs, transcriptomic analysis confirmed that EVs carry oncogenic mRNA and non-coding RNA signatures enriched in migration, cell cycle, and tumor progression genes [42]. EV signatures (EVsig) also show strong positive associations with canonical EMT transcription factors (TWIST1, SNAI1/2), immunosuppressive genes (PD-L1, TGFB1), MMPs, and cancer stem cell markers (CD44, ALDH1A1), suggesting interconnected mechanisms of immune evasion and metastatic capability [28].

Breast cancer cells (MDA-MB-231) were directly treated with HPV DNA-carrying EVs, resulting in enhanced colony formation and invasiveness, which highlights their role in remodeling the tumor microenvironment to support triple-negative breast cancer (TNBC) aggressiveness [43]. Notably, exosomes from HPV-16 E7-expressing non-small cell lung cancer (NSCLC) cells dramatically promoted EMT, evidenced by the upregulation of mesenchymal markers (Vimentin, N-cadherin, Snail1, Slug, Twist1) and suppression of E-cadherin. These effects correlated with elevated EGFR signaling in both cells and exosomes, implicating exosomal EGFR as a mediator of E7-induced EMT. Additionally, 12 differentially expressed miRNAs-such as the upregulated miR-10b-5p, miR-221-3p, and miR-381-3p were linked to EMT progression and metastasis. Pathway analysis of these miRNAs indicated convergence on key oncogenic signaling networks, including FoxO and Hippo pathways, highlighting the regulatory potential of HPV-derived exosomal miRNAs in metastasis [44].

### 2.3. Angiogenesis

**Figure 3:**
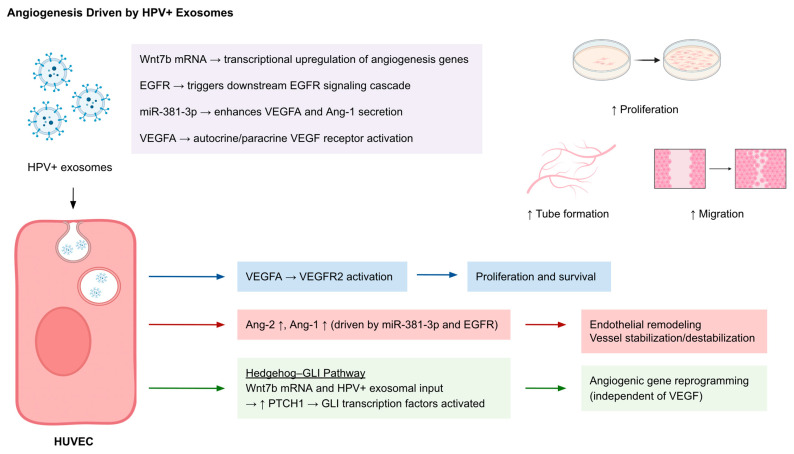
Angiogenesis driven by HPV^+^ exosomes. HPV^+^ exosomes deliver pro-angiogenic cargo including Wnt7b mRNA, EGFR, miR-381-3p, and VEGFA to human umbilical vein endothelial cells (HUVECs). Following uptake, VEGFA activates VEGFR2 signaling to enhance proliferation and survival; miR-381-3p and EGFR upregulate Ang-1 and Ang-2, promoting endothelial remodeling and vessel stabilization/destabilization; and Wnt7b mRNA triggers Hedgehog–GLI signaling (↑ PTCH1), enabling angiogenic gene reprogramming independently of VEGF. These signaling pathways collectively enhance HUVEC proliferation, migration, and tube formation, resulting in vascular remodeling within the tumor microenvironment. Abbreviations: Ang, angiopoietin; EGFR, epidermal growth factor receptor; GLI, glioma-associated oncogene; HPV, human papillomavirus; HUVEC, human umbilical vein endothelial cell; mRNA, messenger RNA; PTCH1, patched homolog 1; VEGF, vascular endothelial growth factor; VEGFR2, vascular endothelial growth factor receptor 2.

Exosomes derived from HPV-positive cancer cells have emerged as effective regulators of tumor angiogenesis by altering endothelial cell behavior through transferring bioactive cargo (Figure 3) (Table 1). Proliferation, tube formation, and migration were enhanced in human umbilical vein endothelial cells (HUVECs) by exosomes from cervical cancer (CC) cells, which were shown to promote angiogenesis. The transfer of Wnt7b mRNA in this case is a partial mediator activating angiogenic signaling in recipient cells [45]. Moreover, HPV(+) exosomes upregulate angiogenesis-related transcription in HUVECs, including VEGF-A, VEGFR2, and angiopoietin-2, while downregulating VEGF-B and angiopoietin-1, which indicates reprogramming of endothelial gene expression. Notably, the observed pro-angiogenic activity was independent of VEGF content and instead correlated with activation of the Hedgehog–GLI pathway, which was indicated by increased PTCH1 levels [46]. Tumor-derived exosomes (TEX) from head and neck squamous cell carcinoma (HNSCC) lines such as PCI-13 and SCCVII induced angiogenic remodeling in HUVECs after rapid internalization, with increased tube formation, proliferation, and migration, along with increased expression of CD31+ endothelial structures and α-SMA^+^ pericyte coverage. These TEXs were enriched with angiogenesis-associated proteins like uPA, VEGF, IGFBP3, MMP-9, and tissue factor [47]. In another model, exosomes secreted by HPV-16 E7-overexpressing non-small cell lung cancer (NSCLC) cells stimulated in vitro and in vivo angiogenesis. The underlying mechanism was attributed to exosomal EGFR and miR-381-3p, both of which promote endothelial secretion of VEGFA and Angiopoietin-1 (Ang-1). The angiogenic effects were significantly reduced by the inhibition of exosome release or EGFR activation, indicating that the E7-induced exosomal content contributes to vascular remodeling [48].

### 2.4. Selective Cargo

In shaping the cell’s behavior and the tumor microenvironment, diverse classes of RNAs, proteins, and even viral DNA, carried in HPV-associated extracellular vesicles (EVs), have various roles as the selective and functionally important molecular cargo (Table 2). Proteomic and transcriptional analysis of EV profiling revealed that the vesicle content, or cargo, is not solely a passive consequence of the cell of origin, but a result of distinct sorting strategies. For example, in HN12-derived EVs (HPV+), RNA-seq analysis showed an oncogenic transcriptome that favored genes regulating cell cycle progression, migration, and tumor development, including SGK1, MAD1L1, and EEF2, all of which correlate with poor patient survival in head and neck squamous cell carcinoma (HNSCC) cohorts. Notably, EV protein and RNA contents only exhibit a partial overlap, implying the presence of a deliberate cargo selection mechanism [42,49].

MicroRNAs (miRNAs) are a critical component of EV-mediated communication. In K49 and K16 cells, differential packaging of miR-17, miR-19a, miR-21, miR-34a, and miR-590-5p into EVs was observed, which were distinct from their intracellular expression patterns, confirming selective export [50]. These miRNAs target cell cycle regulators such as Cyclin D1 (CCND1), CDK4, and p53, which suggests that their removal by exosomes may facilitate cell cycle deregulation in the donor cell while also affecting cells in the tumor milieu like stromal or immune cells. miR-99a-5p, functioning as a tumor suppressor, is also secreted in EVs likely as a survival strategy by reducing growth-inhibitory signals in tumor cells [51]. In addition to miRNAs, long non-coding RNAs (lncRNAs) such as HOTAIR, MALAT1, and MEG3 are highly enriched in HPV(+) EVs. These lncRNAs are known regulators of chromatin dynamics, EMT, and immune evasion. This reinforces the role of exosomes as epigenetic modulators of the TME [52]. HPV oncoproteins in particular can influence both intracellular and exosomal microRNA (miRNA) profiles, contributing to tumor progression. Exosomal miRNAs play a regulatory role in cell proliferation, senescence, and apoptosis. Silencing of HPV18 E6/E7 in HeLa cells altered ten intracellular miRNAs: downregulating pro-proliferative and anti-apoptotic miRNAs such as miR-17-5p, miR-378a-3p, and miR-7-5p, while upregulating tumor-suppressive ones like miR-143-3p, miR-23a-3p, miR-23b-3p, and miR-27b-3p. An exosomal seven-miRNA signature was also induced by E6/E7 silencing, with decreased levels of let-7d-5p, miR-20a-5p, miR-378a-3p, miR-423-3p, miR-7-5p, and miR-92a-3p, and increased miR-21-5p, suggesting a role for HPV oncogenes in modulating exosomal content to support carcinogenesis [53]. Similarly, HPV16 E6/E7 expression in human keratinocytes altered the miRNA content of exosome-enriched extracellular vesicles (Exo-EVs), with the differential expression of 31 miRNAs, including increased miR-222-3p, miR-320a, and miR-378a-3p, which are predicted to inhibit apoptosis and necrosis. This change potentially aids in tumor survival and immune evasion [54]. Also, in HPV+ and HPV- oropharyngeal squamous cell carcinoma (OPSCC) cells, clustering of miRNAs contained in EVs commonly showed positivity for EV markers, but 14 miRNAs were only increased in the HPV+ cells, and 19 miRNAs were enriched only in HPV- cells. EV miRNA signatures are confirmed to reflect the HPV infection status of the tumor [55].

mRNA profiles can also be modified in HPV+ tumors to achieve the desired TME conditions. In HPV-positive head and neck squamous cell carcinoma (HNSCC) cell-derived exosomes, the miRNA and mRNA profiles were reflective of their parent cells, with HPV+ exosomes enriched in HPV16 E6/E7 mRNAs. Immunoregulatory signatures were observed in HPV+ cells: HPV+ exosomes showed higher levels of CDKN2A, EGFR, TNFSF4, and CXCR4, while HPV- exosomes had increased CCND1, DPP4, FAS, and TGFBR2. miRNA cargo was also manipulated by the virus as shown by the unique expression of miR-205-5p in HPV+ exosomes and miR-1972 in HPV-exosomes. The manipulation of exosomal cargo by HPV could strongly affect altered immune responses and lead to varying clinical outcomes. Uniquely expressed exosomal RNAs leading to this could act as HPV-specific clinical markers in HNSCC [34].

HPV(+) EVs also carry proteins associated with cell survival and immune modulation. Markedly, E6/E7 oncoproteins, p16, Rb, and survivin have been detected as exosomal cargo and they could potentially contribute to oncogenic reprogramming of the recipient cells [25]. Proteomic analyses further identified enriched annexins, heat shock proteins, and metabolic enzymes, along with groups of proteins involved in extracellular matrix remodeling, cell adhesion, and immune response [42]. In head and neck cancer (HNC), HPV infection acts to alter the molecular cargo of exosomes. Proteomic and transcriptomic profiling revealed that exosomes from HPV+ cells carry distinct proteins like EIF2AK2, RELA and miRNAs such as miR-130b that are enriched in HPV carcinogenesis-related pathways. The upregulation of miR-130b, accompanied by the downregulation in EIF2AK2 and RELA, reflected HPV-mediated suppression of immune signaling. These three exosomal markers were utilized in a biomarker panel to accurately discriminate between HPV+ HNC patients from HPV- patients and healthy controls, suggesting high diagnostic potential [56]. In addition, in HNSCC cell lines, the protein content of EVs was confirmed to depend on HPV positivity, with 133 proteins present only in EVs from HPV+ cells and 644 only in HPV- cells. Some proteins were only enriched in exosomes compared to whole cell lysates, which shows active and selective packaging processes. Tenascin-C (TNC), HLA-A, E-cadherin, EGFR, EPHA2 and Cytokeratin 19 (CK19) were proposed as strong candidates for EV-based biomarkers in HPV-associated HNSCC [57]. HPV DNA itself has been detected in circulating EVs from patients, including those with triple-negative breast cancer (TNBC). It was also shown to be transferable to HPV-negative fibroblasts in vitro, resulting in induction of proliferative and inflammatory gene expression such as c-Myc, Cyclin D1, IL-6, and CD44 [43]. This suggests a potential mechanism of systemic HPV DNA dissemination via EVs, which could contribute to the oncogenic effects of the virus beyond the primary infection site.

These findings confirm that the EV cargo of HPV-positive cells is selected to enhance tumor progression, immune modulation, and intercellular communication. The presence of viral oncoproteins, immunomodulatory RNAs, and cancer-associated transcripts in EVs makes them key players in HPV-driven carcinogenesis and they seem to be promising targets for biomarker development and therapeutic intervention.

## 3. Exosomes and Liquid Biopsy: Diagnostic and Prognostic Applications

### 3.1. Exosomal Nucleic Acids

**Figure 4:**
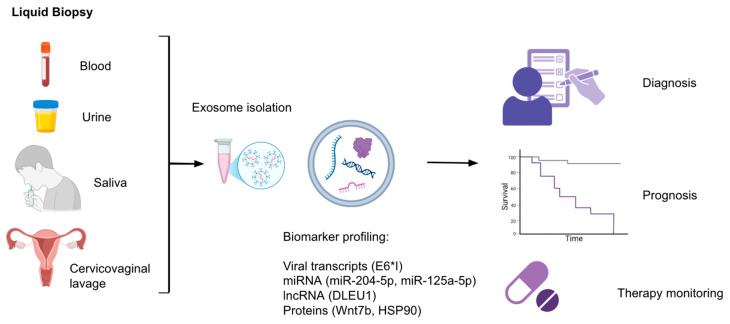
Diagnostic Liquid Biopsy Pipeline Using Exosomes. Exosomes can be isolated from various body fluids, including blood, urine, saliva, and cervicovaginal lavage. Following isolation, biomarker profiling can detect viral transcripts (e.g., E6*I), microRNAs (e.g., miR-204-5p, miR-125a-5p), long non-coding RNAs (e.g., DLEU1), and proteins (e.g., Wnt7b, HSP90). These exosomal components may support diagnosis, provide prognostic information, and enable real-time therapy monitoring. Abbreviations: HPV, human papillomavirus; lncRNA, long non-coding RNA; miRNA, microRNA.

Exosome-based liquid biopsy as an emerging field offers a minimally invasive and impactful approach for early detection, prognosis assessment, and therapy monitoring of HPV-associated cancers (Figure 4). Exosomes contain stable cargo such as miRNAs, lncRNAs, mRNAs, proteins, and even viral transcripts that reflect the changing molecular state of their parent cells, making them valuable sources of biomarkers related to diseases. Multiple studies have identified exosomal microRNAs (exo-miRNAs) as promising diagnostic and prognostic tools (Table 1). For instance, serum exosomal miR-204-5p levels progressively increase across cervical lesion severity (healthy < LSIL < HSIL), suggesting applicability in risk stratification for cervical lesions and disease monitoring [38]. Likewise, plasma levels of EV-associated Wnt7b are significantly elevated in cervical cancer patients and correlate with clinical invasiveness and poor outcomes. EV-Wnt7b serves as an independent prognostic marker and is integrated into a validated survival-predictive framework [45].

HPV-specific transcripts have also been detected in exosomes. The spliced E6*I mRNA variant of HPV16, detectable in HPV16-positive cervical cancer patients from plasma-derived exosomal RNA, supports the use of EV-based RNA profiling for detection of viral oncogenes and disease monitoring [30] (Table 1). Additionally, exosomal miR-99a-5p, which is enriched in the plasma of HPV-positive head and neck squamous cell carcinoma (HNSCC) patients but inversely expressed in tissue, could reflect an export mechanism controlled by tumor cell-specific pathways and emerges as a candidate non-invasive diagnostic biomarker [42,51]. In HPV+ HNSCC, 118 exo-miRNAs showed different expression levels compared to HPV- cells and patterns were detectable in the serum of early-stage HNSCC patients. miR-99a-5p, for example, correlated with recurrence-free survival, showing promise as a biomarker in HPV-related HNSCC [58]. In head and neck cancers, a core set of 25 exo-miRNAs were differentially secreted across all HPV-positive and HPV-negative HNSCC cell lines, including consistently upregulated miR-451a and miR-16-2-3p. These miRNAs were also significantly elevated in serum samples from early-stage patients, showing reproducibility and high clinical relevance [58]. An interesting finding is that some miRNAs like miR-125a-5p and miR-3168 were abundant in serum but absent in tumor cells, hinting at a non-tumor source [58]. The discrepancy between low tumor expression and high serum exosomal levels of miR-125a-5p may suggest secretion by non-tumor cell populations, such as immune or stromal cells, as part of a systemic response to HPV infection or tumorigenesis. This further highlights the complexity of EV profiling in search of potential tumor-related biomarkers. In another cancer type, cervical cancer, exosomal miR-125a-5p levels were found to be significantly lower compared to healthy controls. Notably, HPV+ patients showed even lower levels than HPV- ones. This downregulation in miR-125a-5p is likely shaped by the HPV-related suppression of p53, as HPV has been shown to inhibit expression of miR-125a-5p. The reduction in levels of tumor suppressive miR-125a-5p could have a role in cervical carcinogenesis, which could make it a potential biomarker for diagnosis [59]. In cervicovaginal lavage samples, miR-21 and miR-146a were specifically enriched in the exosomal fraction, and not the supernatant and correlated with CD9 (an exosomal marker) levels. The increase was significant in cervical cancer patients compared to both HPV-positive and HPV-negative controls. HPV infection alone was also a contributor to the upregulation. The active secretion and abnormal elevation of exosome-encapsulated miR-21 and miR-146a by the cancer tissue presents other candidates as non-invasive biomarkers to detect cervical cancer [60].

Even though exosome release may be manipulated in the presence of HPV infection, the change in HPV type may not affect the exosomal content. Exosomal miRNAs like let-7d-3p and miR-30d-5p are valuable diagnostic biomarkers for non-invasive screening of cervical cancer and its precursors, irrespective of HPV type. Expression profiles of 8 miRNA signatures targeting the viral carcinogenesis pathway were also not different among various HPV types [61]. Some non-invasive biomarkers could be applicable for multiple HPV types and provide a tool for broad-spectrum screening, as in this example.

### 3.2. Exosomal Protein Biomarkers

Presence of HPV can also modulate the exosomal protein content, which presents proteins as potential biomarker candidates in discriminating HPV+ patients (Table 1). Proteins like ANXA1, HSP90, and ACTN4 were upregulated in oral cancer derived EVs and have been proposed as markers to assess disease progression [42]. Beyond serving as markers, proteins such as ANXA1 and HSP90 are known to actively modulate tumor–immune interactions; ANXA1 can influence macrophage polarization, while HSP90 acts as a chaperone for oncogenic signaling molecules. Their presence in exosomes suggests a possible role in shaping the tumor microenvironment and promoting immune evasion [62,63,64,65,66]. The modified proteins can also be enzymes related to important pathways. In HPV-driven oropharyngeal cancer (OPC) patients, HPV16 E6/7 DNA was detected in salivary exosomes in 80% but not found in healthy controls. In salivary exosomes that were HPV-modified, six glycolytic enzymes (ALDOA, GAPDH, LDHA, LDHB, PGK1, and PKM) were significantly upregulated. This shows enhanced glycolysis and potentially formation of the Warburg effect in the setting of HPV-driven carcinogenesis. HIF-1α was predicted to get activated and act as an upstream regulator, supporting HPV’s role in metabolic reprogramming. This change in protein profiles of salivary exosomes could be useful in the discrimination of HPV+ OPC patients from healthy individuals [67].

### 3.3. Exosomal Long Non-Coding RNAs

Exosomal long non-coding RNAs (lncRNAs) also present as changing cargo in HPV+ exosomes (Table 3). Particularly HOTAIR, MALAT1, and MEG3 are highly enriched in cervicovaginal lavage (CVL) samples from HPV-positive patients and indicate potential use for early detection and HPV-related risk stratification [52]. Exosomal DLEU1 (a lncRNA) was also significantly upregulated in cervical cancer patients compared to healthy controls and those with cervical intraepithelial neoplasia (CIN). Its expression was associated with tumor burden and poorer prognosis. It was concluded that HPV infection status did not correlate with exosomal DLEU1 levels but previous studies show that DLEU1 expression in cervical cancer tissues was linked to HPV infection [68,69]. This shows that while HPV influences DLEU1 expression of tissues, the exosomal release of the lncRNA is reflective of something more than solely the HPV infection status: malignant progression. The presence of increased exosomal DLEU1 could act as a diagnostic and prognostic biomarker in CC [68].

### 3.4. Monitoring of Treatment Response

EVs also show probability of being real-time indicators of therapy response through longitudinal profiling. In HPV/p16+ HNSCC patients, exosomal miR-21, -let-7a, and -181a were significantly higher than HPV- patients in the initial diagnosis. miR-21 was downregulated in HPV+ tissue but increased in exosomes. In HPV/p16- patients, EV-miR-21 expression was found to significantly increase during the 12-month follow-up. These suggest diagnostic and treatment monitoring utility [70].

Altogether, the stability and accessibility of exosomes, along with their varied content, position them as potentially transformative tools in HPV-related oncology. Their cargo provides a molecular picture of tumor activity and host interactions. This could enable sensitive, real-time assessment of disease state and the therapy response. Continued validation across large, prospective cohorts is likely to make their role clearer in clinical practice for HPV-related cancer types like cervical and head and neck cancers.

### 3.5. Exosomes and Traditional Screening Methods

Current evidence from clinical studies and retrospective analyses provides important context for the potential role of exosomal cargo as a complementary approach to established cancer screening methods. A retrospective analysis of 608 cervical cancer patients showed that cytology tests had an overall detection rate of 68.86%, markedly lower than the 93.17% achieved by tissue biopsy. Recent work using plasma exosomal miRNA profiling identified an eight-miRNA panel with excellent diagnostic accuracy (AUC = 0.992) for distinguishing CIN I– from CIN II+, outperforming individual miRNAs and performing consistently across HPV types [61]. While Pap smear/cytology and HPV DNA testing remain the gold standard in cervical cancer screening, their sensitivity and specificity limitations highlight the potential of liquid biopsy as a minimally invasive, repeatable tool that captures real-time molecular changes. Exosomal miRNAs are stable and abundant in biofluids and reflect tumor-specific expression patterns but further large-scale validation and standardization are needed before they can be integrated into clinical screening alongside traditional methods [71,72,73].

## 4. Exosome-Related Therapies

### 4.1. Exosome-Induced Immune Stimulation

**Figure 5:**
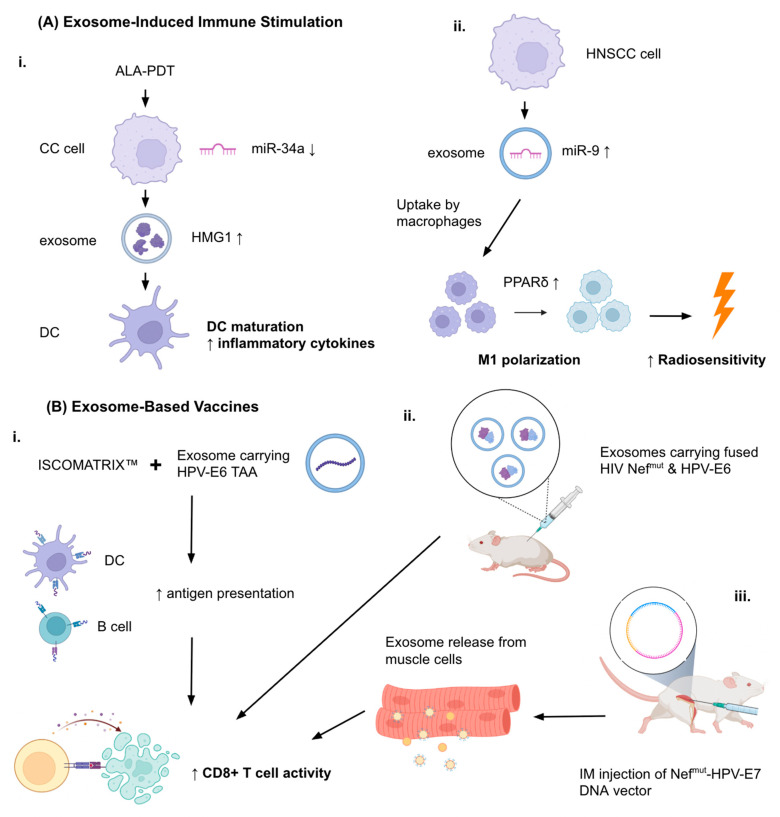

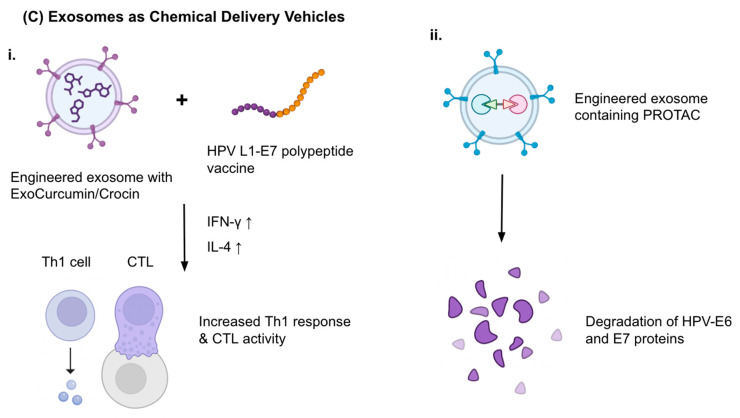
Therapeutic Roles of Exosomes in HPV-Related Cancers. (**A**) Immune stimulation via exosome cargo—example mechanisms include (i) 5-aminolevulinic acid photodynamic therapy (ALA-PDT) enhancing dendritic cell (DC) maturation through exosomal HMGB1 release, and (ii) HPV+ HNSCC-derived exosomal miR-9 promoting macrophage M1 polarization and radiosensitivity. (**B**) Exosome-based vaccines—Engineered exosomes carrying HPV E6/E7 tumor-associated antigens (TAAs) combined (i) with adjuvants (e.g., ISCOMATRIX™) or (ii,iii) Nefmut-based fusion systems elicit robust cytotoxic T lymphocyte (CTL) responses, preventing or suppressing tumor growth in preclinical models. (**C**) Drug delivery and targeted degradation strategies—examples include (i) exosomes loaded with natural compounds (ExoCurcumin, ExoCrocin) co-administered with HPV L1-E7 vaccines to boost Th1/CTL immunity and (ii) engineered exosomes delivering proteolysis-targeting chimeras (PROTACs) for E6/E7 degradation, potentially enhanced by ligand modification for tumor-specific targeting. Abbreviations: ALA-PDT, 5-aminolevulinic acid photodynamic therapy; CC, cervical cancer; CTL, cytotoxic T lymphocyte; DC, dendritic cell; EGFR, epidermal growth factor receptor; HNSCC, head and neck squamous cell carcinoma; HPV, human papillomavirus; PROTAC, proteolysis-targeting chimera; TAA, tumor-associated antigen; Th1, T helper type 1.

Therapies based on exosomes are emerging as potential tools in the treatment of HPV-associated cancers (Table 4). Multiple studies show their ability to induce antitumor immunity, be used as delivery agents for therapeutic molecules, and help overcome tumor resistance mechanisms. Jin et al. (2018) demonstrated that the 5-aminolevulinic acid photodynamic therapy (ALA-PDT) could suppress the growth of HPV+ CC cells by promoting apoptosis and downregulating miR-34a (Figure 5A) [74]. This led to increased HMGB1 secretion in exosomes of the CC cells. The change in exosomal content supplemented DC maturation and the production of pro-inflammatory cytokines, leading to strengthened anti-tumor immune response. These effects could be reversed either by silenced HMGB1 or restoration of miR-34a, which confirms that the miR-34a/HMGB1/exosome axis is therapeutically relevant. The ALA-PDT treatment was confirmed to stimulate the immune response through its actions of exosomal mechanisms specifically for HPV+ CC. Additionally, Tong et al. (2020) showed that HPV+ HNSCC exosomes carry an enriched amount of miR-9 [40]. Macrophages in the surrounding TME could import these exosomes which resulted in the downregulation of PPARδ and the subsequent M1 polarization of the macrophages. Interestingly, HPV+ HNSCC cells having a high number of pro-inflammatory M1 macrophages exhibited augmented radiosensitivity when treated with 6 Gy ionizing radiation, according to the increase in γ-H2AX foci. The authors propose miR-9 as a potential treatment element in HPV+ HNSCC (Figure 5A) [40].

### 4.2. Exosome-Based Vaccines

Another study related to HPV immunity was from Manfredi et al. (2016) who investigated the treatment efficiency of exosomes engineered to carry HPV-E6 tumor-associated antigen (TAA) in combination with the ISCOMATRIX™ adjuvant (containing purified ISCOPREP^TM^ saponin, cholesterol, and phospholipids) (Figure 5B) [77]. They found that the co-administration of engineered exosomes carrying HPV-E6 tumor-associated antigen (TAA) and the adjuvant molecule resulted in significantly enhanced in vitro cross-presentation of antigens by B-lymphoblastoid and immature dendritic cells. In in vivo experiments, the CD8+ T cell response against the HPV E6 protein was seen to exceed the one with the presence of exosomes alone. Exosome vaccines enhanced with adjuvant molecules like ISCOMATRIX™ could show promise as immune response-enhancing therapies [77].

In a study by di Bonito et al. (2015), a novel exosome-based vaccination strategy was generated to create CD8+ T lymphocyte (CTL) responses against specific protein antigens [75]. The setup used a mutated HIV-1 Nef protein (Nef^mut^) as the exosome-anchoring domain. HPV E7 was fused to Nef^mut^, which enabled efficient loading of the antigen into the exosomes. Even without including the fusogenic protein VSV-G, the engineered exosomes showed immunogenicity same as lentiviral virus-like particles (VLPs). Exosomes carrying HPV E7 could induce E7-targeted CTL responses successfully in mouse models also. When administered prophylactically, this method prevented tumor growth and therapeutically suppressed the established tumors. So, Nef^mut^-fused E7 loaded onto exosomes were found to be an effective method to elicit CTL immunity (Figure 5B) [75]. In a following study by di Bonito et al. (2017), a strong CD8+ T cell immune response against the HPV E7 DNA vector was generated through endogenous production of engineered exosomes, in an effort to create an alternative to in vitro-engineered exosomes that posed problems in terms of clinical applicability [76]. A DNA vector for E7 and Nef^mut^ were fused together and injected intramuscularly, resulting in the production of exosomes and their release into the circulation. In a TC-1 tumor mouse model, antitumor effects were observed as a result of the cytotoxic response (Figure 5B) [76]. This cost-effective and scalable method could supplement immunotherapy platforms in HPV-related cancers.

### 4.3. Exosomes as Drug Delivery Vehicles

Exosomes were also studied as carriers for chemotherapeutic molecules such as natural compounds or synthetic proteins. Abbasifarid et al. (2021) investigated the immunological and tumor-therapeutic effects of exosomes loaded with crocin and curcumin compounds administered in combination with a synthetic HPV L1-E7 polypeptide vaccine, in C57BL/6 mouse models bearing TC-1 tumors [78]. In certain doses, the ExoCrocin and ExoCurcumin were not cytotoxic and could enter tumor cells. Cytokine assays showed the secretion of IFN-γ and IL-4 was increased in L1-E7 polypeptide + ExoCurcumin/ExoCrocin groups compared to controls. IFN-γ levels were higher in the L1-E7 polypeptide + ExoCurcumin/ExoCrocin groups compared to both ExoCurcumin/ExoCrocin or the polypeptide alone. The coadministration of the engineered exosomes and the polypeptide vaccine produced a significant level of immunity directed toward Th1 response and CTL activity, demonstrating potential as a successful immune-focused therapy candidate. (Figure 5C) [78].

### 4.4. Engineered Exosomes: PROTACs and Targeting Strategies

As for synthetic exosomal therapeutic cargo, proteolysis-targeting chimeras (PROTACs) present as new candidates. Although ongoing studies are present in various types of cancers it is an understudied area in HPV-related cancer therapy [80]. Exosomes are derived from body fluids and engineered to carry PROTACs through methods such as liposome fusion and electroporation. The PROTACs facilitate E3 ubiquitin ligase activity to degrade E6 and E7 proteins, which are critical elements of HPV-related carcinogenesis (Figure 5C). This allows for targeting HPV-associated mechanisms and elimination of non-specific toxicity. In addition, exosomes can be specifically targeted to tissues by modifying surface ligands [79]. This has emerged as a promising strategy to improve tumor-specific accumulation and reduce off-target effects. The approach involves functionalizing the exosomal membrane with ligands that bind selectively to receptors overexpressed on tumor cells or within the tumor microenvironment [81]. For example, folate-conjugated exosomes have been developed to target folate receptor-α, which is frequently upregulated in various epithelial cancers, including certain cervical cancer subtypes [82,83]. RGD (arginine–glycine–aspartic acid) peptide-modified exosomes can bind integrins αvβ3 and αvβ5, which are enriched on angiogenic tumor vasculature and some HPV-associated tumor cells [84,85]. In another approach, EGFR-targeting exosomes have been generated by displaying anti-EGFR nanobodies or peptides on their surface, enabling selective delivery to EGFR-overexpressing head and neck squamous cell carcinoma cells [86]. Functionalization can be achieved through genetic engineering, where exosomal membrane proteins such as Lamp2b or CD63 are fused to targeting ligands, or through chemical conjugation where ligands are covalently linked to exosomal lipids or proteins after isolation. In addition to improving tumor targeting, ligand-modified exosomes can potentially enhance intracellular delivery by exploiting receptor-mediated endocytosis, supplementing the bioavailability and therapeutic efficacy of encapsulated agents [81,87]. This strategy aims to strengthen tumor suppressor pathways by eliminating oncogenic driver proteins and may prove to be successful but is a relatively new area especially in HPV-related cancers. Adaptation could be possible for HPV-positive cancers by selecting ligands that recognize receptors specifically upregulated in HPV-driven tumors, but there is much work to be done for clinical translation.

### 4.5. Exosomes and Therapy Resistance

In addition to being used as therapeutic agents, exosomes produced by cancer cells can contribute to treatment resistance mechanisms (Table 5). In their review on therapy targets for HPV+/− oral and tongue cancers, Gupta et al. (2021) present exosomes from cancer stem cells (CSC) as important agents in therapy resistance, recurrence, and metastasis [88]. In tongue squamous cell carcinoma (TSCC), sometimes associated with HPV positivity, one proposed mechanism of mediating tumor progression and relapse is through the CSC-derived exosomes that transmit miRNAs associated with chemoresistance. Targeting of these vesicles is discussed as a route to overcome relapse and resistance in TSCC. [88]. In an investigation of the reasons for failure of anti-PD-1 immunotherapy (nivolumab) in HNSCC, Hill et al. (2023) also point out cargo of exosomes [89]. Non-responders to therapy had high levels of IL-8 and reduced levels of IL-8 targeting miRNAs in their exosomes like miR-146a. Dsg2, an oncoprotein, was found to be elevated in HPV+ tumors and was present in high levels in non-responders’ post-treatment. The data indicate that Dsg2 regulates IL-8 indirectly by suppressing miR-146a, which normally targets IL-8 transcripts for downregulation. This suppression is likely post-transcriptional, as miR-146a acts after IL-8 mRNA synthesis, reducing its stability or translation. High Dsg2 in HPV+ non-responders therefore maintain low miR-146a, allowing sustained IL-8 production via NF-κB activation, promoting immune suppression. The Dsg2/miR-146a/IL-8 axis is defined as a potential contributor to immune checkpoint inhibitor resistance and could be targeted to improve therapy outcomes [89,90].

Taken together, these findings demonstrate that exosomes could have various therapeutic applications in HPV-associated cancers, from immune stimulation and tumor antigen presentation to targeted delivery of therapeutic agents and enhancement of peptide-based vaccines. The mentioned studies and reviews each show a unique aspect of exosome biology that can be utilized as a treatment strategy for immunotherapy, combination therapies, or next-generation vaccines. While promising, findings also highlight the need for further validation and optimization to overcome the remaining challenges in clinical translation.

## 5. Future Directions

Despite the growing interest in exosomes as diagnostic and therapeutic agents in HPV-associated cancers, several important challenges remain. First, there is a need to standardize exosome isolation, quantification, and characterization protocols [91,92]. In the mentioned studies, patient exosomes are derived from different sources: body fluids like blood plasma, saliva, urine, and from cervicovaginal lavage (Figure 1) [13,30,51,60,93,94]. Blood plasma is the most preferred, but there is no comparison between the effectiveness of different sources and how their utility could differ, and for each study, the most convenient source is chosen for analysis. Methodological consistency is essential to ensure reproducibility and clinical translatability. The Minimal Information for Studies of Extracellular Vesicles (MISEV) guidelines, developed by the International Society for Extracellular Vesicles, provide standardized criteria for EV isolation, characterization, and reporting [95,96]. Applying these recommendations is particularly important in the context of HPV-driven malignancies, where biomarker studies often vary widely in sample type, EV isolation method, and analytical approach. By adhering to MISEV, researchers can generate more comparable datasets, facilitate meta-analyses, and accelerate the translation of exosome research into reliable diagnostic and prognostic tools for cervical and other HPV-associated cancers. In addition, no consensus is present on which exosomal markers need to be checked for HPV infection characterization or the related tumor stage/behavior determination. Because exosomal cargo is very heterogeneous and closely dependent on the cellular context, singular isolation of specific biomolecules is hard [97,98]. Without standardization of the isolation and analysis processes, clinical reproducibility and approval will remain elusive.

Recent evidence also highlights the importance of considering the molecular mechanisms mediated by HPV-associated exosomes when designing future studies. Exosomes derived from HPV(+) cancers carry immunomodulatory molecules such as PD-L1, TGF-β, CD47, and miR-1468-5p, which contribute to immune evasion by suppressing cytotoxic T cell activity and promoting lymphangiogenesis [27,28,29,30,31,32,33]. At the same time, unique exosomal cargo in HPV+ tumors may partly explain why they remain more responsive to immunotherapy compared to HPV− cancers [25,34,35]. Elucidating these dual immunological effects could help refine exosome-based biomarkers for predicting treatment response and immune checkpoint efficacy.

Exosomes are also active drivers of epithelial–mesenchymal transition (EMT) and metastasis in HPV-related cancers. Cargo such as EGFR, TWIST1, SNAI1/2, and pro-metastatic miRNAs (e.g., miR-10b-5p, miR-221-3p) promote motility, invasion, and extracellular matrix remodeling [27,42,44]. Similarly, exosome-mediated angiogenesis, involving molecules like Wnt7b mRNA, VEGFA, and miR-381-3p, supports tumor vascularization and progression [45,46,47,48]. These mechanistic insights suggest that future therapeutic strategies could focus on inhibiting the release, uptake, or downstream signaling of pro-metastatic and pro-angiogenic exosomes.

Selective cargo packaging into HPV+ exosomes, including viral oncogenes (E6/E7), oncogenic mRNAs (EGFR, TP53), and regulatory non-coding RNAs such as HOTAIR and MALAT1, indicates that exosome content is not random but purposefully directed to favor tumor growth and immune evasion [34,42,49,50,51,52,53,54,55,56,57]. Future work dissecting the molecular machinery behind cargo selection may pave the way for both engineered therapeutic exosomes and the disruption of tumor-promoting exosome biogenesis.

Biomarker validation across large, diverse, and longitudinal HPV+ cohorts is also lacking. Many studies report exosomal cargo like miRNAs or proteins as promising biomarkers, but few have demonstrated prognostic or predictive strength in real-world clinical settings. Future efforts should focus on integrating liquid biopsy platforms with multi-omics analysis and clinical outcome data to assess utility in early detection, risk stratification, and real-time therapy monitoring [98].

In addition, the therapeutic potential of engineered exosomes, especially those delivering HPV oncoprotein-targeting PROTACs or vaccine antigens, remains promising but underexplored [99]. The biocompatibility of engineered exosomes remains questionable, which is why some researchers try to achieve in vivo-production of therapeutic exosomes. Future preclinical work should emphasize in vivo efficacy, immune profiling (to make sure the exosomes do not activate unwanted host immune reactions), biodistribution, and scalability of the manufacturing process. The development of targeted delivery systems, perhaps via ligand engineering or nanomolecule incorporation, may further enhance precision.

In prospective clinical studies, exosome-mediated mechanisms of therapy resistance and immune evasion such as the IL-8/miR-146a axis should be investigated, particularly in the context of immune checkpoint blockade failure [89]. There is also a lack of studies investigating how exosomes are affected during administration of other kinds of mainstream or preclinical chemotherapeutic drugs. Understanding the resistance pathways governed by exosomes could lead to the development of predictive markers using liquid biopsy data or combination strategies to restore immune responsiveness when faced with resistance in cancer treatment. Molecular mechanisms underlying resistance should be further explored in basic studies before clinical application.

## 6. Conclusions

Even though there are various in vitro studies investigating the relationship between HPV infection, related cancers and the role of extracellular vesicles/exosomes, there is not an extensive amount of research focused on the diagnostic and therapeutic purposes of exosomes in cancers specifically associated with HPV [99]. Because the relationship between EV/exosomal content and HPV infections remains a mystery, and new findings are coming to light every day, it is not possible to extend the basic research into the clinical application area yet. Much work has to be done to solidify the related molecular pathways in HPV related cancers and how EVs are affected by them or used as tools by the tumor cells to supplement cancer progression [97]. Then, in addition to introducing engineered exosomes as therapeutic vehicles, the organic EVs can also potentially be manipulated to increase the therapeutic efficiency or as a treatment method itself.

With the rapid advancement of exosome research and biotechnology, the coming decade may see the integration of exosomal diagnostics and therapeutics into the routine clinical framework for HPV-related cancers, provided that these translational and regulatory obstacles are actively addressed [100].

## Figures and Tables

**Table 1:** Functional Roles of HPV^+^ Exosomes in Immune Modulation, EMT/Metastasis, and Angiogenesis.

Mechanism	Exosomal Cargo	Source	Target/Pathway	Functional Outcome
Immune Modulation	miR-1468-5p	Cervical cancer exosomes	LECs → HMBOX1/SOCS1 suppression → JAK2/STAT3 activation	↑ PD-L1, ↑ lymphangiogenesis, impaired CD8^+^ T cell activity, poor survival
	PD-L1, IL-6, VEGFA	HPV^+^ exosomes	T cells and TME	Immunosuppression
	CD47 (EGFR/STAT3-dependent packaging)	HPV^+^ exosomes	Monocytes	↓ Phagocytosis (“don’t eat me” signal)
	CXCL10 → fibroblast PD-L1 exosomes	HPV^+^ cervical cancer cells	CAFs via JAK1/STAT1	Immunosuppressive feedback loop
	Viral RNAs (E6/E7), EGFR, miR-205-5p vs. MUC-1, HLA-DRA, miR-1972	HPV^+^ vs. HPV^−^ HNSCC exosomes	DCs	HPV^+^: promote DC maturation; HPV^−^: inhibit antigen presentation
	miR-204-5p	HPV16 E6^+^ cervical cancer exosomes	Macrophages	JAK2 suppression → M2 polarization
	miR-9	HPV^+^ HNSCC exosomes	Macrophages	STAT1/NF-κB/AP-1 activation → iNOS, TNF-α, IL-6 ↑ → M1 polarization
	lncRNAs (Treg-derived exosomes)	HPV^+^ penile SCC	CD8^+^ T cells	↑ MAL → CTL apoptosis
EMT & Metastasis	PRSS56, ALPL ↑; ITGB4, TACSTD2, S100A6 ↓	HeLa EVs	Adhesion and migration pathways	Disrupted adhesion, EMT-like shift
	TWIST1, SNAI1/2, PD-L1, CD44, ALDH1A1	HN12 EVs	EMT TFs and stemness genes	EMT induction, immune evasion, CSC phenotype
	HPV DNA	EVs to TNBC (MDA-MB-231)	Breast cancer cells	↑ Colony formation, invasion
	EGFR, miR-10b-5p, miR-221-3p, miR-381-3p	HPV16 E7^+^ NSCLC	FoxO/Hippo pathways	EMT progression, invasion, metastasis
Angiogenesis	Wnt7b mRNA	Cervical cancer exosomes	Endothelial cells	↑ Tube formation, ↑ migration
	VEGFA, VEGFR2, Ang-2 ↑, Ang-1 ↓	HPV^+^ exosomes	Hedgehog–GLI pathway	Endothelial reprogramming, angiogenesis independent of VEGF
	VEGF, IGFBP3, MMP-9, TF, uPA	HNSCC TEXs (PCI-13, SCCVII)	HUVECs	↑ Endothelial proliferation, CD31^+^ vessels, α-SMA^+^ pericytes
	EGFR, miR-381-3p	HPV16 E7^+^ NSCLC	EGFR pathway	VEGFA & Ang-1 secretion → angiogenesis in vitro/in vivo

HPV^+^ exosomes modulate the tumor microenvironment (TME) through immune evasion (PD-L1, CD47, miR-1468-5p), EMT and metastasis (EGFR, TWIST1, miR-10b-5p), and angiogenesis (Wnt7b mRNA, VEGFA, miR-381-3p). Abbreviations: CAF, cancer-associated fibroblast; CTL, cytotoxic T lymphocyte; DC, dendritic cell; EMT, epithelial–mesenchymal transition; HPV, human papillomavirus; TME, tumor microenvironment; VEGF, vascular endothelial growth factor.

**Table 2:** Selective molecular cargo in HPV^+^ exosomes.

Cargo Type	Representative Cargo	Mechanism/Effect	Clinical Implication
miRNAs	miR-17, miR-21, miR-590-5p (oncogenic); miR-99a-5p (tumor suppressor exported)	Cell cycle deregulation, apoptosis inhibition; removal of suppressors enhances tumor survival	Biomarkers, tumor survival strategy
lncRNAs	HOTAIR, MALAT1, MEG3	Epigenetic reprogramming, EMT induction, immune evasion	Potential therapeutic targets for chromatin modulation
mRNAs	HPV16 E6/E7, SGK1, CXCR4, EGFR	Oncogenic transcriptome enrichment, immune regulation	Prognostic markers correlate with poor survival in HNSCC
Proteins	E6/E7, p16, Rb, Survivin, EGFR, TNC, HLA-A, CK19	Immune suppression, ECM remodeling, adhesion regulation	EV-based biomarkers for HPV-related cancers
HPV DNA	Circulating EVs containing HPV DNA (e.g., in TNBC)	Transfer to fibroblasts → ↑ Cyclin D1, c-Myc, IL-6, CD44 expression	Potential systemic oncogenic dissemination via EVs

HPV^+^ exosomes selectively enrich oncogenic miRNAs (miR-17, miR-21), lncRNAs (HOTAIR, MALAT1), mRNAs (E6/E7, EGFR), proteins (Survivin, TNC, CK19), and even HPV DNA, while exporting tumor-suppressive miRNAs (e.g., miR-99a-5p). This cargo contributes to cell cycle deregulation, EMT, immune evasion, and systemic viral dissemination. Abbreviations: CK, cytokeratin; EMT, epithelial–mesenchymal transition; EGFR, epidermal growth factor receptor; HPV, human papillomavirus; lncRNA, long non-coding RNA; miRNA, microRNA; TNC, tenascin-C; VEGF, vascular endothelial growth factor.

**Table 3:** Exosomal Biomarkers in HPV-Associated Cancers.

Biomarker	Cancer Type	HPV Status Association	Clinical Utility	Reference
miRNA:				
miR-21 miR-146a	Cervical Cancer	Upregulated in HPV+ EVs	Diagnosis	Liu et al., 2014 [60]
let-7d-3p miR-30d-5p	Cervical Cancer	Upregulated in HPV+ Evs, regardless of HPV type	Non-invasive screening of CC, diagnosis	Zheng et al., 2019 [61]
miR-125a-5p	Cervical Cancer	Downregulated in HPV+ Evs	Diagnosis	Aixia LV et al., 2021 [59]
miR-451a miR-16-2-3p	HNSCC	Upregulated in HPV+ Evs	Diagnosis, clinical reproducibility	Galiveti et al., 2022 [58]
miR-99a-5p	HNSCC	Enriched in HPV+ plasma Evs	Diagnosis, RFS prediction	Huang et al., 2022 [51] Leung et al., 2021 [42] Galiveti et al., 2022 [58]
miR-21 miR-let-7a miR-181a	HNSCC	Upregulated in HPV+ Evs	Diagnosis, follow-up	Apeltrath et al., 2024 [70]
miR-204-5p	Cervical Cancer	Upregulated in HPV+ CC Evs	Lesion severity stratification, disease monitoring	Chen et al., 2024 [38]
Viral RNA (mRNA):				
HPV16 E6*I	Cervical Cancer	Present in HPV16+ EVs	Viral oncogene detection	Bhat et al., 2022 [30]
DNA:				
HPV16 E6/7 DNA	OPC	Present in HPV16+ salivary EVs	Detection of HPV+ OPC patients	Tang et al., 2021 [67]
Proteins:				
Wnt7b	Cervical Cancer	Elevated in HPV+ CC	Prognosis (OS, RFS)	Qiu et al., 2020 [45]
ANXA1 HSP90 ACTN4	Oral Cancer	Upregulated in HPV+ EVs	Disease progression	Leung et al., 2021 [42]
Glycolytic enzymes (ALDOA, GAPDH, LDHA, LDHB, PGK1, PKM)	OPC	Present in HPV+ salivary Evs	Detection of HPV+ OPC patients	Tang et al., 2021 [67]
lncRNAs:				
HOTAIR MALAT1 MEG3	Cervical Cancer	Enriched in exosomes from CVL samples of HPV+ patients	Early detection, risk stratification	Zhang et al., 2016 [52]
DLEU1	Cervical Cancer	Not HPV-type specific	Tumor burden, prognosis	Chen et al., 2025 [68]

Exosomal biomarkers identified in HPV-associated cancers, grouped by biomolecule type. Abbreviations: CC, cervical cancer; CVL, cervicovaginal lavage; EV, extracellular vesicle; HNSCC, head and neck squamous cell carcinoma; OPC, oropharyngeal carcinoma; OS, overall survival; RFS, recurrence-free survival.

**Table 4:** Engineered Exosome-Based Therapeutics.

Strategy	Cargo	Target Mechanism	Cancer Type/Model	Therapeutic Outcome	Reference
Nef^mut^-HPV E7 Exosomes	E7 fusion protein	CTL generation	Mouse TC-1 tumor	Anti-tumor CTL response	Di Bonito et al., 2015 [75]
DNA vector for E7-Nef^mut^	Endogenous E7 exosome production	Immunization without ex vivo engineering	Mouse TC-1 tumor	Anti-tumor CTL response	Di Bonito et al., 2017 [76]
Exo + ISCOMATRIX™	HPV E6 protein	Enhance antigen presentation	C57 Bl/6 mice	Anti-tumor CTL response	Manfredi et al., 2016 [77]
ExoCurcumin/Crocin + L1-E7 vaccine	Natural compounds + vaccine	Th1/CTL immunity induction	Mouse TC-1 tumor	Increased IFN-γ & IL-4	Abbasifarid et al., 2021 [78]
Exosomal PROTACs	E6/E7 degraders	Oncoprotein elimination	Theoretical model (HPV-related)	Targeted degradation	Mukherjee et al., 2024 [79]

Examples include exosomes carrying HPV E7 fusion proteins to stimulate cytotoxic T lymphocyte (CTL) responses, endogenous production of E7-containing exosomes via DNA vectors, and antigen-loaded exosomes combined with adjuvants to enhance immune activation. Natural compound–loaded exosomes can potentiate vaccine-induced immunity, while proteolysis-targeting chimera (PROTAC)-loaded exosomes aim to selectively degrade HPV oncoproteins. Abbreviations: CTL, cytotoxic T lymphocyte; E6/E7, HPV oncoproteins E6 and E7; HPV, human papillomavirus; IFN-γ, interferon gamma; IL-4, interleukin 4; PROTAC, proteolysis-targeting chimera; Th1, T helper type 1.

**Table 5:** Exosome-Mediated Therapy Resistance Mechanisms.

Mechanism	Exosomal Component	Cancer Type	Effect on Therapy	Reference
Immune checkpoint failure	miR-146a (↓)	HPV+ HNSCC	Dsg2 (↑) IL-8 (↑) Anti-PD-1 resistance	Hill et al., 2023 [89]
Chemoresistance, relapse	miRNA from CSC-derived exosomes	TSCC (HPV+)	miRNA-driven resistance	Gupta et al., 2021 [88]

Exosomal cargo can modulate immune checkpoint efficacy or promote chemoresistance, contributing to treatment failure and tumor relapse. Abbreviations: CSC, cancer stem cell; Dsg2, desmoglein-2; HNSCC, head and neck squamous cell carcinoma; HPV, human papillomavirus; IL-8, interleukin 8; miRNA, microRNA; PD-1, programmed cell death protein 1; TSCC, tongue squamous cell carcinoma.

## Data Availability

No new data were created or analyzed in this study. Data sharing is not applicable to this article.

## Abbreviations

The following abbreviations are used in this manuscript:

ALA-PDT	5-aminolevulinic acid photodynamic therapy
CC	Cervical cancer
CSC	Cancer stem cell
CTL	Cytotoxic T lymphocyte
EV	Extracellular vesicle
HNSCC	Head and neck squamous cell carcinoma
HPV	Human papillomavirus
OPC	Oropharyngeal carcinoma
PROTAC	Proteolysis-targeting chimera
TME	Tumor microenvironment
TSC	Tongue squamous cell carcinoma

## References

[1] Centers for Disease Control and Prevention Cancers Caused by HPV. https://www.cdc.gov/hpv/about/cancers-caused-by-hpv.html.

[2] (2024). World Health Organization Cervical Cancer. https://www.who.int/news-room/fact-sheets/detail/cervical-cancer.

[3] Bray F., Laversanne M., Sung H., Ferlay J., Siegel R.L., Soerjomataram I., Jemal A. (2024). Global Cancer Statistics 2022: GLOBOCAN Estimates of Incidence and Mortality Worldwide for 36 Cancers in 185 Countries. CA Cancer J. Clin..

[4] Baba S.K., Alblooshi S.S.E., Yaqoob R., Behl S., Al Saleem M., Rakha E.A., Malik F., Singh M., Macha M.A., Akhtar M.K. (2025). Human Papilloma Virus (HPV) Mediated Cancers: An Insightful Update. J. Transl. Med..

[5] Berman T.A., Schiller J.T. (2017). Human Papillomavirus in Cervical Cancer and Oropharyngeal Cancer: One Cause, Two Diseases. Cancer.

[6] Yugawa T., Kiyono T. (2009). Molecular Mechanisms of Cervical Carcinogenesis by High-Risk Human Papillomaviruses: Novel Functions of E6 and E7 Oncoproteins. Rev. Med. Virol..

[7] Joura E.A., Giuliano A.R., Iversen O.-E., Bouchard C., Mao C., Mehlsen J., Moreira E.D., Ngan Y., Petersen L.K., Lazcano-Ponce E. (2015). A 9-Valent HPV Vaccine against Infection and Intraepithelial Neoplasia in Women. N. Engl. J. Med..

[8] Rosenblum H.G., Lewis R.M., Gargano J.W., Querec T.D., Unger E.R., Markowitz L.E. (2022). Human Papillomavirus Vaccine Impact and Effectiveness Through 12 Years After Vaccine Introduction in the United States, 2003 to 2018. Ann. Intern. Med..

[9] Branda F., Pavia G., Ciccozzi A., Quirino A., Marascio N., Gigliotti S., Matera G., Romano C., Locci C., Azzena I. (2024). Human Papillomavirus (HPV) Vaccination: Progress, Challenges, and Future Directions in Global Immunization Strategies. Vaccines.

[10] Kuang L., Wu L., Li Y. (2025). Extracellular Vesicles in Tumor Immunity: Mechanisms and Novel Insights. Mol. Cancer.

[11] Chen Y.F., Luh F., Ho Y.S., Yen Y. (2024). Exosomes: A Review of Biologic Function, Diagnostic and Targeted Therapy Applications, and Clinical Trials. J. Biomed. Sci..

[12] Colombo M., Raposo G., Théry C. (2014). Biogenesis, Secretion, and Intercellular Interactions of Exosomes and Other Extracellular Vesicles. Annu. Rev. Cell Dev. Biol..

[13] Li M., Zeringer E., Barta T., Schageman J., Cheng A., Vlassov A.V. (2014). Analysis of the RNA Content of the Exosomes Derived from Blood Serum and Urine and Its Potential as Biomarkers. Philos. Trans. R. Soc. B Biol. Sci..

[14] Li S., Yi M., Dong B., Tan X., Luo S., Wu K. (2021). The Role of Exosomes in Liquid Biopsy for Cancer Diagnosis and Prognosis Prediction. Int. J. Cancer.

[15] Yáñez-Mó M., Siljander P.R.M., Andreu Z., Zavec A.B., Borràs F.E., Buzas E.I., Buzas K., Casal E., Cappello F., Carvalho J. (2015). Biological Properties of Extracellular Vesicles and Their Physiological Functions. J. Extracell. Vesicles.

[16] Wang X., Qiao D., Chen L., Xu M., Chen S., Huang L., Wang F., Chen Z., Cai J., Fu L. (2019). Chemotherapeutic Drugs Stimulate the Release and Recycling of Extracellular Vesicles to Assist Cancer Cells in Developing an Urgent Chemoresistance. Mol. Cancer.

[17] Adams E., Sepich-Poore G.D., Miller-Montgomery S., Knight R. (2022). Using All Our Genomes: Blood-Based Liquid Biopsies for the Early Detection of Cancer. View.

[18] Sachan P., Singh M., Patel M., Sachan R. (2018). A Study on Cervical Cancer Screening Using Pap Smear Test and Clinical Correlation. Asia-Pac. J. Oncol. Nurs..

[19] Zampaoglou E., Boureka E., Gounari E., Liasidi P.-N., Kalogiannidis I., Tsimtsiou Z., Haidich A.-B., Tsakiridis I., Dagklis T. (2025). Screening for Cervical Cancer: A Comprehensive Review of Guidelines. Cancers.

[20] Nikanjam M., Kato S., Kurzrock R. (2022). Liquid Biopsy: Current Technology and Clinical Applications. J. Hematol. Oncol..

[21] Sadri Nahand J., Moghoofei M., Salmaninejad A., Bahmanpour Z., Karimzadeh M., Nasiri M., Mirzaei H.R., Pourhanifeh M.H., Bokharaei-Salim F., Mirzaei H. (2020). Pathogenic Role of Exosomes and MicroRNAs in HPV-Mediated Inflammation and Cervical Cancer: A Review. Int. J. Cancer.

[22] Paskeh M.D.A., Entezari M., Mirzaei S., Zabolian A., Saleki H., Naghdi M.J., Sabet S., Khoshbakht M.A., Hashemi M., Hushmandi K. (2022). Emerging Role of Exosomes in Cancer Progression and Tumor Microenvironment Remodeling. J. Hematol. Oncol..

[23] Lorenc T., Klimczyk K., Michalczewska I., Słomka M., Kubiak-Tomaszewska G., Olejarz W. (2020). Exosomes in Prostate Cancer Diagnosis, Prognosis and Therapy. Int. J. Mol. Sci..

[24] Wang M., Ji S., Shao G., Zhang J., Zhao K., Wang Z., Wu A. (2018). Effect of Exosome Biomarkers for Diagnosis and Prognosis of Breast Cancer Patients. Clin. Transl. Oncol..

[25] Ludwig S., Sharma P., Theodoraki M.N., Pietrowska M., Yerneni S.S., Lang S., Ferrone S., Whiteside T.L. (2018). Molecular and Functional Profiles of Exosomes from HPV(+) and HPV(−) Head and Neck Cancer Cell Lines. Front. Oncol..

[26] Gabaran S.G., Ghasemzadeh N., Rahnama M., Karatas E., Akbari A., Rezaie J. (2025). Functionalized Exosomes for Targeted Therapy in Cancer and Regenerative Medicine: Genetic, Chemical, and Physical Modifications. Cell Commun. Signal..

[27] Acevedo-Sánchez V., Martínez-Ruiz R.S., Aguilar-Ruíz S.R., Torres-Aguilar H., Chávez-Olmos P., Garrido E., Baltiérrez-Hoyos R., Romero-Tlalolini M.d.l.A. (2023). Quantitative Proteomics for the Identification of Differentially Expressed Proteins in the Extracellular Vesicles of Cervical Cancer Cells. Viruses.

[28] Kallinger I., Rubenich D.S., Głuszko A., Kulkarni A., Spanier G., Spoerl S., Taxis J., Poeck H., Szczepański M.J., Ettl T. (2023). Tumor Gene Signatures That Correlate with Release of Extracellular Vesicles Shape the Immune Landscape in Head and Neck Squamous Cell Carcinoma. Clin. Exp. Immunol..

[29] Zhou C., Wei W., Ma J., Yang Y., Liang L., Zhang Y., Wang Z., Chen X., Huang L., Wang W. (2021). Cancer-Secreted Exosomal MiR-1468-5p Promotes Tumor Immune Escape via the Immunosuppressive Reprogramming of Lymphatic Vessels. Mol. Ther..

[30] Bhat A., Yadav J., Thakur K., Aggarwal N., Chhokar A., Tripathi T., Singh T., Jadli M., Veerapandian V., Bharti A.C. (2022). Transcriptome Analysis of Cervical Cancer Exosomes and Detection of HPVE6*I Transcripts in Exosomal RNA. BMC Cancer.

[31] Ludwig S., Marczak L., Sharma P., Abramowicz A., Gawin M., Widlak P., Whiteside T.L., Pietrowska M. (2019). Proteomes of Exosomes from HPV(+) or HPV(−) Head and Neck Cancer Cells: Differential Enrichment in Immunoregulatory Proteins. Oncoimmunology.

[32] Ke X., Li L., Yan Q., Wang X., Liu P. (2025). EGFR/STAT3 Signaling Mediates the Upregulation of CD47 in HPV-Positive Cervical Cancer by Activating P65 and Exosome Transporter RAB31. Neoplasma.

[33] Chen X., He H., Xiao Y., Hasim A., Yuan J., Ye M., Li X., Hao Y., Guo X. (2021). CXCL10 Produced by HPV-Positive Cervical Cancer Cells Stimulates Exosomal PDL1 Expression by Fibroblasts via CXCR3 and JAK-STAT Pathways. Front. Oncol..

[34] Ludwig S., Sharma P., Wise P., Sposto R., Hollingshead D., Lamb J., Lang S., Fabbri M., Whiteside T.L. (2020). Mrna and Mirna Profiles of Exosomes from Cultured Tumor Cells Reveal Biomarkers Specific for Hpv16-Positive and Hpv16-Negative Head and Neck Cancer. Int. J. Mol. Sci..

[35] Wang B., Zhang S., Tong F., Wang Y., Wei L. (2022). HPV+ HNSCC-Derived Exosomal MiR-9-5p Inhibits TGF-β Signaling-Mediated Fibroblast Phenotypic Transformation through NOX4. Cancer Sci..

[36] Li Z., Chen B., Han H., Xue T., Li X. (2025). Effect of Exosome-Derived LncRNA on MAL^+^ CTL Apoptosis in Remodeling of the Immune Microenvironment in HPV^+^ Penile Squamous Cell Carcinoma. J. Clin. Oncol..

[37] Zhang G., Liao Y., Pan X., Zhang X. (2022). Exosomes from HPV-16 E7-Pulsed Dendritic Cells Prevent the Migration, M1 Polarization, and Inflammation of Macrophages in Cervical Cancer by Regulating Catalase 2 (CAT2). Ann. Transl. Med..

[38] Chen X., Liu Y., Luo X., Pan T., Zhang T., Hu L., Wu B., Liu W., Wei F. (2024). HPV16 E6-Induced M2 Macrophage Polarization in the Cervical Microenvironment via Exosomal MiR-204-5p. Sci. Rep..

[39] Yadav J., Tripathi T., Chaudhary A., Janjua D., Joshi U., Aggarwal N., Chhokar A., Keshavam C.C., Senrung A., Bharti A.C. (2025). Influence of Head and Neck Cancer Exosomes on Macrophage Polarization. Cytokine.

[40] Tong F., Mao X., Zhang S., Xie H., Yan B., Wang B., Sun J., Wei L. (2020). HPV + HNSCC-Derived Exosomal MiR-9 Induces Macrophage M1 Polarization and Increases Tumor Radiosensitivity. Cancer Lett..

[41] Iuliano M., Mangino G., Chiantore M.V., Zangrillo M.S., Accardi R., Tommasino M., Fiorucci G., Romeo G. (2018). Human Papillomavirus E6 and E7 Oncoproteins Affect the Cell Microenvironment by Classical Secretion and Extracellular Vesicles Delivery of Inflammatory Mediators. Cytokine.

[42] Leung L.L., Riaz M.K., Qu X., Chan J., Meehan K. (2021). Profiling of Extracellular Vesicles in Oral Cancer, from Transcriptomics to Proteomics. Semin. Cancer Biol..

[43] De Carolis S., Storci G., Ceccarelli C., Savini C., Gallucci L., Sansone P., Santini D., Seracchioli R., Taffurelli M., Fabbri F. (2019). HPV DNA Associates With Breast Cancer Malignancy and It Is Transferred to Breast Cancer Stromal Cells by Extracellular Vesicles. Front. Oncol..

[44] Zhou Z., Wu X., Zhan R., Li X., Cheng D., Chen L., Wang T., Yu H., Zhang G., Tang X. (2022). Exosomal Epidermal Growth Factor Receptor Is Involved in HPV-16 E7-Induced Epithelial-Mesenchymal Transition of Non-Small Cell Lung Cancer Cells: A Driver of Signaling In Vivo. Cancer Biol. Ther..

[45] Qiu J., Sun S., Tang X., Lin Y., Hua K. (2020). Extracellular Vesicular Wnt7b Mediates HPV E6-Induced Cervical Cancer Angiogenesis by Activating the β-Catenin Signaling Pathway. J. Exp. Clin. Cancer Res..

[46] Bhat A., Yadav J., Thakur K., Aggarwal N., Tripathi T., Chhokar A., Singh T., Jadli M., Bharti A.C. (2021). Exosomes from Cervical Cancer Cells Facilitate Pro-Angiogenic Endothelial Reconditioning through Transfer of Hedgehog–GLI Signaling Components. Cancer Cell Int..

[47] Ludwig N., Yerneni S.S., Razzo B.M., Whiteside T.L. (2018). Exosomes from HNSCC Promote Angiogenesis through Reprogramming of Endothelial Cells. Mol. Cancer Res..

[48] Zhan R., Yu H., Zhang G., Ding Q., Li H., Li X., Tang X. (2024). Exosomal EGFR and MiR-381-3P Mediate HPV-16 E7 Oncoprotein-Induced Angiogenesis of Non-Small Cell Lung Cancer. Front. Biosci.-Landmark.

[49] Yadav J., Chaudhary A., Tripathi T., Janjua D., Joshi U., Aggarwal N., Chhokar A., Keshavam C.C., Senrung A., Bharti A.C. (2024). Exosomal Transcript Cargo and Functional Correlation with HNSCC Patients’ Survival. BMC Cancer.

[50] Chiantore M.V., Iuliano M., Mongiovì R.M., Dutta S., Tommasino M., Di Bonito P., Accardi L., Mangino G., Romeo G. (2022). The E6 and E7 Proteins of Beta3 Human Papillomavirus 49 Can Deregulate Both Cellular and Extracellular Vesicles-Carried MicroRNAs. Infect. Agents Cancer.

[51] Huang Q., Shen Y.J., Hsueh C.Y., Zhang Y.F., Yuan X.H., Zhou Y.J., Li J.Y., Lin L., Wu C.P., Hu C.Y. (2022). Plasma Extracellular Vesicles-Derived MiR-99a-5p: A Potential Biomarker to Predict Early Head and Neck Squamous Cell Carcinoma. Pathol. Oncol. Res..

[52] Zhang J., Liu S.C., Luo X.H., Tao G.X., Guan M., Yuan H., Hu D.K. (2016). Exosomal Long Noncoding RNAs Are Differentially Expressed in the Cervicovaginal Lavage Samples of Cervical Cancer Patients. J. Clin. Lab. Anal..

[53] Honegger A., Schilling D., Bastian S., Sponagel J., Kuryshev V., Sültmann H., Scheffner M., Hoppe-Seyler K., Hoppe-Seyler F. (2015). Dependence of Intracellular and Exosomal MicroRNAs on Viral E6/E7 Oncogene Expression in HPV-Positive Tumor Cells. PLoS Pathog..

[54] Harden M.E., Munger K. (2017). Human Papillomavirus 16 E6 and E7 Oncoprotein Expression Alters MicroRNA Expression in Extracellular Vesicles. Virology.

[55] Peacock B., Rigby A., Bradford J., Pink R., Hunter K., Lambert D., Hunt S. (2018). Extracellular Vesicle MicroRNA Cargo Is Correlated with HPV Status in Oropharyngeal Carcinoma. J. Oral. Pathol. Med..

[56] Qin T.X., Ng W.H., Chek M.F., Tang K.D. (2025). Proteomic and Transcriptomic Profiling in Exosomes Derived from HPV-Positive Head and Neck Cancer. Biochem. Biophys. Res. Commun..

[57] Goudsmit C., da Veiga Leprevost F., Basrur V., Peters L., Nesvizhskii A., Walline H. (2021). Differences in Extracellular Vesicle Protein Cargo Are Dependent on Head and Neck Squamous Cell Carcinoma Cell of Origin and Human Papillomavirus Status. Cancers.

[58] Galiveti C.R., Kuhnell D., Biesiada J., Zhang X., Kelsey K.T., Takiar V., Tang A.L., Wise-Draper T.M., Medvedovic M., Kasper S. (2023). Small Extravesicular MicroRNA in Head and Neck Squamous Cell Carcinoma and Its Potential as a Liquid Biopsy for Early Detection. Head Neck.

[59] Lv A., Tu Z., Huang Y., Lu W., Xie B. (2020). Circulating Exosomal MiR-125a-5p as a Novel Biomarker for Cervical Cancer. Oncol. Lett..

[60] Liu J., Sun H., Wang X., Yu Q., Li S., Yu X., Gong W. (2014). Increased Exosomal MicroRNA-21 and MicroRNA-146a Levels in the Cervicovaginal Lavage Specimens of Patients with Cervical Cancer. Int. J. Mol. Sci..

[61] Zheng M., Hou L., Ma Y., Zhou L., Wang F., Cheng B., Wang W., Lu B., Liu P., Lu W. (2019). Exosomal Let-7d-3p and MiR-30d-5p as Diagnostic Biomarkers for Non-Invasive Screening of Cervical Cancer and Its Precursors. Mol. Cancer.

[62] Liu S. (2025). MiR-374a/b-5p Suppresses Cell Growth in Papillary Thyroid Carcinoma Through Blocking Exosomal ANXA1-Induced Macrophage M2 Polarization. Biochem. Genet..

[63] Leung C.O., Gu C.Y., Lee T.K. (2025). Abstract 4405: Annexin A1 Regulates Lenvatinib Resistance through Mediating STAT3/S100A6 Signaling Cascade and M2 Macrophage Polarization in Hepatocellular Carcinoma. Cancer Res..

[64] Zheng Y., Jiang H., Yang N., Shen S., Huang D., Jia L., Ling J., Xu L., Li M., Yu K. (2024). Glioma-Derived ANXA1 Suppresses the Immune Response to TLR3 Ligands by Promoting an Anti-Inflammatory Tumor Microenvironment. Cell Mol. Immunol..

[65] Wickenberg M., Mercier R., Yap M., Walker J., Baker K., LaPointe P. (2024). Hsp90 Inhibition Leads to an Increase in Surface Expression of Multiple Immunological Receptors in Cancer Cells. Front. Mol. Biosci..

[66] Hong L., Tanaka M., Yasui M., Hara-Chikuma M. (2024). HSP90 Promotes Tumor Associated Macrophage Differentiation during Triple-Negative Breast Cancer Progression. Sci. Rep..

[67] Tang K.D., Wan Y., Zhang X., Bozyk N., Vasani S., Kenny L., Punyadeera C. (2021). Proteomic Alterations in Salivary Exosomes Derived from Human Papillomavirus-Driven Oropharyngeal Cancer. Mol. Diagn. Ther..

[68] Chen Y., Cui F., Wu X., Zhao W., Xia Q. (2025). The Expression and Clinical Significance of Serum Exosomal-Long Non-Coding RNA DLEU1 in Patients with Cervical Cancer. Ann. Med..

[69] Dong S., Zhang Y., Wang Y. (2023). Role of Extracellular Vesicle in Human Papillomavirus-Associated Cervical Cancer. J. Cancer Res. Clin. Oncol..

[70] Apeltrath C., Simon F., Riders A., Rudack C., Oberste M. (2024). Extracellular Vesicle MicroRNAs as Possible Liquid Biopsy Markers in HNSCC—A Longitudinal, Monocentric Study. Cancers.

[71] Martinelli C., Ercoli A., Vizzielli G., Burk S.R., Cuomo M., Satasiya V., Kacem H., Braccia S., Mazzarotti G., Miriello I. (2025). Liquid Biopsy in Gynecological Cancers: A Translational Framework from Molecular Insights to Precision Oncology and Clinical Practice. J. Exp. Clin. Cancer Res..

[72] Kepsha M.A., Timofeeva A.V., Chernyshev V.S., Silachev D.N., Mezhevitinova E.A., Sukhikh G.T. (2024). MicroRNA-Based Liquid Biopsy for Cervical Cancer Diagnostics and Treatment Monitoring. Int. J. Mol. Sci..

[73] Jiang J., Li L., Zhang C., Yang C., Dai Y., Chen Y., Huang Y., Xie L., Xiang Y., Yuan J. (2025). The Application of Liquid Biopsy Techniques in Cervical Cancer Diagnosis, Prediction and Therapeutic Surveillance. J. Gynecol. Oncol..

[74] Jin Y., Guan Z., Wang X., Wang Z., Zeng R., Xu L., Cao P. (2018). ALA-PDT Promotes HPV-Positive Cervical Cancer Cells Apoptosis and DCs Maturation via MiR-34a Regulated HMGB1 Exosomes Secretion. Photodiagn. Photodyn. Ther..

[75] Di Bonito P., Ridolfi B., Columba-Cabezas S., Giovannelli A., Chiozzini C., Manfredi F., Anticoli S., Arenaccio C., Federico M. (2015). HPV-E7 Delivered by Engineered Exosomes Elicits a Protective CD8+ T Cell-Mediated Immune Response. Viruses.

[76] Di Bonito P., Chiozzini C., Arenaccio C., Anticoli S., Manfredi F., Olivetta E., Ferrantelli F., Falcone E., Ruggieri A., Federico M. (2017). Antitumor HPV E7-Specific CTL Activity Elicited by in Vivo Engineered Exosomes Produced through DNA Inoculation. Int. J. Nanomed..

[77] Manfredi F., di Bonito P., Ridolfi B., Anticoli S., Arenaccio C., Chiozzini C., Morelli A.B., Federico M. (2016). The CD8+ T Cell-Mediated Immunity Induced by HPV-E6 Uploaded in Engineered Exosomes Is Improved by ISCOMATRIXTM Adjuvant. Vaccines.

[78] Abbasifarid E., Bolhassani A., Irani S., Sotoodehnejadnematalahi F. (2021). Synergistic Effects of Exosomal Crocin or Curcumin Compounds and HPV L1-E7 Polypeptide Vaccine Construct on Tumor Eradication in C57BL/6 Mouse Model. PLoS ONE.

[79] Mukerjee N., Maitra S., Ghosh A. (2024). Exosome-Based Therapy and Targeted PROTAC Delivery: A New Nanomedicine Frontier for HPV-Mediated Cervical Cancer Treatment. Clin. Transl. Discov..

[80] Wang C., Zhang Y., Chen W., Wu Y., Xing D. (2024). New-Generation Advanced PROTACs as Potential Therapeutic Agents in Cancer Therapy. Mol. Cancer.

[81] Liu Q., Li D., Pan X., Liang Y. (2023). Targeted Therapy Using Engineered Extracellular Vesicles: Principles and Strategies for Membrane Modification. J. Nanobiotechnol..

[82] Zhu L., Dong D., Yu Z.L., Zhao Y.F., Pang D.W., Zhang Z.L. (2017). Folate-Engineered Microvesicles for Enhanced Target and Synergistic Therapy toward Breast Cancer. ACS Appl. Mater. Interfaces.

[83] Zhang G., Liu F., Jia E., Jia L., Zhang Y. (2016). Folate-Modified, Cisplatin-Loaded Lipid Carriers for Cervical Cancer Chemotherapy. Drug Deliv..

[84] Danhier F., Le Breton A., Préat V. (2012). RGD-Based Strategies to Target Alpha(v) Beta(3) Integrin in Cancer Therapy and Diagnosis. Mol. Pharm..

[85] Gu Y., Du Y., Jiang L., Tang X., Li A., Zhao Y., Lang Y., Liu X., Liu J. (2022). Avβ3 Integrin-Specific Exosomes Engineered with Cyclopeptide for Targeted Delivery of Triptolide against Malignant Melanoma. J. Nanobiotechnol..

[86] Kooijmans S.A.A., Aleza C.G., Roffler S.R., van Solinge W.W., Vader P., Schiffelers R.M. (2016). Display of GPI-Anchored Anti-EGFR Nanobodies on Extracellular Vesicles Promotes Tumour Cell Targeting. J. Extracell. Vesicles.

[87] Yang Q., Li S., Ou H., Zhang Y., Zhu G., Li S., Lei L. (2024). Exosome-Based Delivery Strategies for Tumor Therapy: An Update on Modification, Loading, and Clinical Application. J. Nanobiotechnol..

[88] Gupta S., Kumar P., Das B.C. (2021). HPV+ve/−ve Oral-Tongue Cancer Stem Cells: A Potential Target for Relapse-Free Therapy. Transl. Oncol..

[89] Hill B.L., Calder A.N., Flemming J.P., Guo Y., Gilmore S.L., Trofa M.A., Daniels S.K., Nielsen T.N., Gleason L.K., Antysheva Z. (2023). IL-8 Correlates with Nonresponse to Neoadjuvant Nivolumab in HPV Positive HNSCC via a Potential Extracellular Vesicle MiR-146a Mediated Mechanism. Mol. Carcinog..

[90] Wang F., Sun T., Wang N., Wei W., Mei Y., Yan Q. (2025). DSG2 Promotes Pancreatic Cancer Stem Cell Maintenance via Support of Tumour and Macrophage Cellular Cross-Talk. Cell Death Dis..

[91] Bastón E., García-Agulló J., Peinado H. (2025). The Influence of Extracellular Vesicles on Tumor Evolution and Resistance to Therapy. Physiol. Rev..

[92] Mivehchi H., Eskandari-Yaghbastlo A., Emrahoglu S., Masouleh S.S., Faghihinia F., Ayoubi S., Afjadi M.N. (2025). Tiny Messengers, Big Impact: Exosomes Driving EMT in Oral Cancer. Pathol. Res. Pract..

[93] Tang S., Cheng H., Zang X., Tian J., Ling Z., Wang L., Xu W., Jiang J. (2025). Small Extracellular Vesicles: Crucial Mediators for Prostate Cancer. J. Nanobiotechnol..

[94] Zhang R., Su J., Xue S.-L., Yang H., Ju L.-L., Ji Y., Wu K.-H., Zhang Y.-W., Zhang Y.-X., Hu J.-F. (2016). HPV E6/P53 Mediated down-Regulation of MiR-34a Inhibits Warburg Effect through Targeting LDHA in Cervical Cancer. Am. J. Cancer Res..

[95] Welsh J.A., Goberdhan D.C.I., O’Driscoll L., Buzas E.I., Blenkiron C., Bussolati B., Cai H., Di Vizio D., Driedonks T.A.P., Erdbrügger U. (2024). Minimal Information for Studies of Extracellular Vesicles (MISEV2023): From Basic to Advanced Approaches. J. Extracell. Vesicles.

[96] Saint-Pol J., Culot M. (2025). Minimum Information for Studies of Extracellular Vesicles (MISEV) as Toolbox for Rigorous, Reproducible and Homogeneous Studies on Extracellular Vesicles. Toxicol. Vitr..

[97] Tang L., Zhang W., Qi T., Jiang Z., Tang D. (2025). Exosomes Play a Crucial Role in Remodeling the Tumor Microenvironment and in the Treatment of Gastric Cancer. Cell Commun. Signal..

[98] Shan Z., Su X., Liu L., Duan S. (2025). Liquid Biopsy Based on EV Biomarkers: A New Frontier for Early Diagnosis and Prognosis Assessment of Cancer at ESMO 2024. Nano TransMed.

[99] Owliaee I., Khaledian M., Boroujeni A.K., Shojaeian A. (2023). Engineered Small Extracellular Vesicles as a Novel Platform to Suppress Human Oncovirus-Associated Cancers. Infect. Agent Cancer.

[100] Semeradtova A., Liegertova M., Herma R., Capkova M., Brignole C., Del Zotto G. (2025). Extracellular Vesicles in Cancer´s Communication: Messages We Can Read and How to Answer. Mol. Cancer.

